# Preclinical evaluation of puerarin for the treatment of non-alcoholic fatty liver disease: a systematic review and meta-analysis

**DOI:** 10.3389/fphar.2026.1795374

**Published:** 2026-04-13

**Authors:** Jingya Li, Meng Li, Ruotong Meng, Siying An, Xin Zhang, Zixuan Shang, Yufei Chen, Yiming Qi, Chunzhi Su

**Affiliations:** 1 Hebei University of Chinese Medicine, Shijiazhuang, Hebei, China; 2 First Affiliated Hospital of Hebei University of Chinese Medicine, Shijiazhuang, Hebei, China

**Keywords:** animal model, mechanism of action, meta-analysis, non-alcoholic fatty liver disease, puerarin

## Abstract

**Background:**

Kudzu root is commonly prescribed in traditional Chinese medicine for non-alcoholic fatty liver disease (NAFLD). Its key active ingredient, puerarin, has shown promising protective effects in experimental NAFLD models. Yet the overall efficacy and the main biological mechanisms have not been systematically assessed.

**Objective:**

This meta-analysis will synthesize evidence from animal studies to determine how puerarin influences liver-related outcomes in NAFLD. It will also summarize the mechanistic pathways that may account for these effects.

**Method:**

Literature retrieval was performed up to September 2025, encompassing eight databases. The risk of bias in the included studies was evaluated using the SYRCLE assessment tool. Data analyses were then performed in Stata 15. The primary outcome included triglycerides (TG), total cholesterol (TC), low-density lipoprotein cholesterol (LDL-C), high-density lipoprotein cholesterol (HDL-C), alanine aminotransferase (ALT), and aspartate aminotransferase (AST). Secondary outcome comprised interleukin-6 (IL-6), interleukin-1β (IL-1β), tumor necrosis factor-alpha (TNF-α), superoxide dismutase (SOD), malondialdehyde (MDA), and glutathione peroxidase (GSH-Px). Subgroup analyses, publication bias assessments, and sensitivity analyses were performed to ensure the robustness.

**Results:**

20 studies were included, involving 331 animals. Puerarin treatment significantly reduced levels of TG, TC, and LDL-C, while improving HDL-C in animal models of NAFLD. Additionally, puerarin significantly upregulated antioxidant indicators SOD and GSH-Px and lowered MDA levels. Furthermore, it inhibited the expression of inflammatory factors IL-6, IL-1β, and TNF-α.

**Conclusion:**

This meta-analysis concludes that puerarin demonstrates substantial liver-protective effects in NAFLD models by regulating lipid metabolism, anti-inflammatory, and antioxidant. Collectively, these finding lays a solid evidence-based foundation for the subsequent advancement of clinical translation research and the in-depth exploration of its underlying mechanisms.

**Systematic Review Registration:**

https://www.crd.york.ac.uk/prospero/, Identifier CRD420251170089.

## Introduction

Non-alcoholic fatty liver disease (NAFLD) is a chronic liver condition linked closely to obesity, insulin resistance, and metabolic syndrome, characterized by diffuse hepatic steatosis in individuals with no significant alcohol intake or other identifiable liver disease causes (Liang et al., 2025). Since June 2023, NAFLD has been redefined as metabolic dysfunction-associated fatty liver disease (MASLD). To maintain alignment with the terminology used in the included trials and prior publications, this systematic review and meta-analysis retains the term NAFLD throughout (Zhu et al., 2025). In its initial stages, NAFLD presents as hepatic steatosis due to excessive fat accumulation in hepatocytes, potentially progressing to non-alcoholic steatohepatitis, liver fibrosis, cirrhosis, and even hepatocellular carcinoma (Liu et al., 2025). In recent years, NAFLD has surpassed other chronic liver conditions to impact 30.05% of the global population (Younossi et al., 2023), emerging as the most prevalent worldwide (Al Kazman et al., 2025). By 2050, the projected number of new NAFLD cases among the global population aged 15 to 49 is estimated to reach 48.29 million, with associated deaths expected to increase to 23,396.5 (Wang M. et al., 2025). NAFLD not only presents a significant public health threat but also imposes a substantial socioeconomic burden, highlighting it as an urgent global public health challenge (Wu et al., 2025). Nonetheless, to date, no highly effective and safe drugs for NAFLD have received approval (Asghari et al., 2025). Consequently, comprehending the pathogenesis of NAFLD and identifying and developing innovative therapeutic approaches and drug interventions stand as critical objectives in contemporary hepatology (Xu et al., 2022).

Current NAFLD management primarily emphasizes lifestyle interventions, focusing on dietary adjustments and regular exercise. Positive outcomes are only evident in patients who strictly adhere to these changes (Paternostro and Trauner, 2022; Rong et al., 2023). Previous studies have shown high dropout rates among patients following lifestyle modifications, resulting in suboptimal final outcomes and raising concerns about the long-term feasibility and effectiveness of such interventions (Hernandez Roman and Patel, 2020). In the pharmacological treatment of NAFLD, statins can mitigate liver damage by reducing lipotoxicity, while metformin and thiazolidinediones enhance insulin sensitivity and decrease hepatic lipid synthesis (Attia et al., 2021). Notably, in 2024, the U.S. Food and Drug Administration (FDA) granted accelerated approval to Rezdiffra as a targeted therapy for NAFLD (Hasan et al., 2025). Longer-term studies are still needed to clarify the durability of these findings and determine their broader clinical relevance. (Bittla et al., 2024). Although these drugs can effectively treat NAFLD, their utilization is hindered by side effects, significant limitations, and poor patient compliance (Khazaei-Poul et al., 2025).

Internationally recognized as a complementary and alternative therapy, traditional Chinese medicine demonstrates pharmacologic effects characterized by multiple components, targets, and pathways. It underscores the therapeutic principles of a “holistic perspective” and “syndrome differentiation and treatment” focusing on individual variations and the multifactorial etiology of diseases, aligning well with the intricate pathogenesis of NAFLD (Zhang et al., 2025a). Natural herbal medicines have now emerged as a crucial resource for contemporary drug development, offering unique chemical structures that present novel targets, propelling advancements in clinical research for human disease treatment (Newman and Cragg, 2016). Consequently, the urgent imperative in the current medical landscape is the identification of natural bioactive compounds with superior efficacy, enhanced safety, and the potential for intervening in NAFLD (Zhou et al., 2021).

**FIGURE 1:**
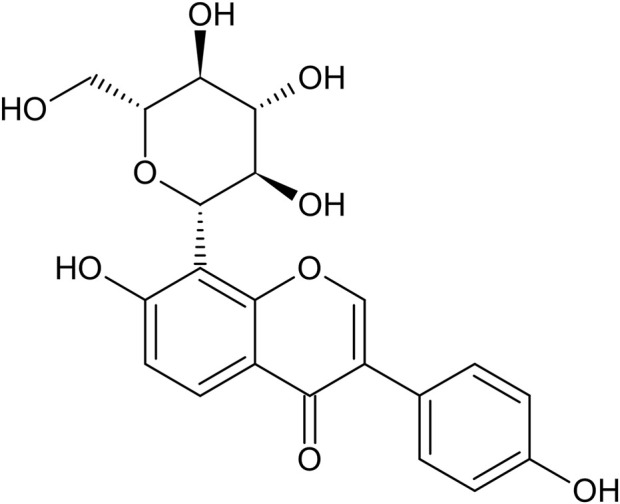
The chemical structure of puerarin.

The dried root of the wild kudzu or sweet kudzu vine (Pueraria lobata), known as kudzu root, is commonly utilized in TCM for various therapeutic purposes. It is recognized for its abilities to relieve muscle tension, reduce fever, promote fluid production, quench thirst, facilitate rash eruption, lift yang energy to alleviate diarrhea, ease spasms, and counteract alcohol toxicity (Chen et al., 2023). Puerarin (Figure 1), the principal active compound derived from Pueraria lobata roots, consists of small molecules like flavonoids, triterpenoids, and coumarins, along with larger molecules such as polysaccharides and proteins (Kou et al., 2025). Previous studies have demonstrated that puerarin possesses pharmacological properties such as antioxidant, anti-inflammatory, and insulin-sensitizing effects (Zhou et al., 2014; Zeng et al., 2018). Recently, researchers have begun to investigate the potential applications of puerarin within the digestive system, particularly focusing on its role in alleviating the symptoms of NAFLD (Kou et al., 2025).

Evidence suggests that an antioxidant fraction derived from kudzu root (Antioxidant Pueraria lobata, APL) attenuates oxidative stress through activation of the Keap1–Nrf2–HO-1 signaling axis, thereby limiting hepatic lipid deposition and ameliorating NAFLD. This study not only provides scientific evidence for the medicinal development of kudzu root but also presents a novel approach for natural drug therapy in NAFLD (Dong et al., 2024). Furthermore, experiments have confirmed the functional properties of kudzu root protein; by activating the Nrf2/Keap1 signaling pathway, it enhances the activity of superoxide dismutase and peroxidase while increasing GSH-Px levels. It markedly alleviates oxidative injury in HepG2 cells, reducing post-insult increases in reactive oxygen species (ROS) and malondialdehyde (MDA) after oxidative stress (Pang et al., 2023). Recent studies have elucidated the mechanism by which puerarin affects NAFLD through the gut-liver axis. Puerarin mitigates hepatic lipid accumulation and inflammatory infiltration in mice by enhancing short-chain fatty acid levels via gut microbiota modulation and by repairing the intestinal mucosal barrier. Additionally, it activates hepatic mitochondrial autophagy and modulates the mRNA expression of lipid metabolism genes to alleviate NAFLD (Sun et al., 2025). Multiple studies using animal models have shown that puerarin confers protection against NAFLD (Li Z. et al., 2024; Fang et al., 2024; Yang et al., 2024). However, these studies assessed the efficacy of puerarin within narrowly defined experimental frameworks. They exhibited heterogeneity in modeling conditions and observational parameters, and discussions regarding relevant mechanisms remained fragmented, lacking a systematic cognitive framework (Zhou et al., 2022). By synthesizing these recent findings, we aim to provide a more comprehensive understanding of the mechanism of action of puerarin, thereby reinforcing its therapeutic potential in NAFLD.

Current research on puerarin primarily emphasizes preclinical studies. While considerable evidence supports its beneficial effects on NAFLD, the conclusions arise from experiments conducted across various laboratories, leading to discrepancies in outcomes. Consequently, it is essential to adopt standardized methods to synthesize these research findings.

Current research on puerarin primarily emphasizes preclinical studies. While considerable evidence supports its beneficial effects on NAFLD, the conclusions arise from experiments conducted across various laboratories, leading to discrepancies in outcomes. Consequently, it is essential to adopt standardized methods to synthesize these research findings. Systematic reviews, as a secondary research methodology, synthesize a variety of findings to address specific research questions. They integrate multiple datasets to increase sample sizes and enhance statistical power, while also systematically incorporating studies and rigorously evaluating bias to mitigate its effects and reduce unnecessary animal use. Developing an evidence-informed structure for animal studies can strengthen the bridge between preclinical results and their eventual implementation in clinical practice.

Until now, no research has delivered an extensive synthesis of rigorous, evidence-based medical information nor conducted thorough examinations of the pharmacological mechanisms by which puerarin impacts NAFLD. This study aims to gather preclinical research data on puerarin, conduct a systematic review and meta-analysis, clarify its hepatoprotective effects in animal models of NAFLD, and elucidate the underlying mechanisms.

## Materials and methods

### Systematic review registration

The execution of this systematic review and meta-analysis fully complied with the protocol registered in PROSPERO (CRD420251170089).

### Search strategy

Relevant literature was identified by screening eight databases from their origin through September 2025. This search strategy utilized medical subject headings and free-text terms, including “Puerarin,” “Kudzu,” “Kudzu root extract puerarin,” “Non-alcoholic Fatty Liver Disease,” “Nonalcoholic Fatty Liver Disease,” “NAFLD,” “Fatty Liver, Nonalcoholic,” “Fatty Livers, Nonalcoholic,” “Liver, Nonalcoholic Fatty,” “Livers, Nonalcoholic Fatty,” “Nonalcoholic Fatty Liver,” “Nonalcoholic Fatty Livers,” “Nonalcoholic Steatohepatitis,” “Steatohepatitides, Nonalcoholic,” and “Steatohepatitis, Nonalcoholic.” For detailed search strategies, refer to the Supplementary Material.

### Inclusion and exclusion criteria

The selection of eligible studies was determined by the PICOS framework, with strict adherence to predefined inclusion criteria: (P) involvement of NAFLD animal models; (I) use of puerarin in the treatment group; (C) control group with no puerarin treatment or only lifestyle modifications; (O) studies were required to evaluate one or more of the specified efficacy endpoints: TC, TG, LDL-C, HDL-C, ALT, AST, and markers of inflammatory or oxidative stress; (S) the study type was limited to preclinical research. The exclusion criteria were defined as follows: 1) non-animal studies, specifically excluding human subjects, *ex vivo* experiments, and publication types like reviews and meeting proceedings; 2) articles for which the original text was unavailable; 3) studies lacking a distinct comparator arm or involving polypharmaceutical interventions; 4) duplicate publications; 5) studies that did not provide essential data, such as sample size and standard deviation.

### Study selection and data extraction

Two independent researchers conducted the literature search, selected studies, extracted data, and assessed quality based on predefined inclusion and exclusion criteria. Disagreements were resolved through discussion or by consulting the corresponding author. For data extraction, the same two reviewers independently collected the following information: 1) publication year and first author’s name; 2) detailed description of experimental animals, including species, sex, number, and body weight; 3) method of NAFLD model induction; 4) intervention strategies for experimental and control groups, including administration method, dosage, and treatment duration; and 5) measurement of outcome measures. For studies that present results solely in graphical format, we sought to obtain the datasets through direct communication with the study creators. If no response is received, WebPlotDigitizer 4.5 will be utilized to extract the data. To ensure data accuracy, two reviewers independently performed chart digitization while simultaneously conducting cross-checks. Resolution of extraction errors was achieved by either mutual agreement among reviewers or verification from the relevant institutions.

### Bias risk

The research team performed an independent evaluation utilizing the SYRCLE bias risk assessment tool, formulated by the Center for Research in Animal Intervention and specifically tailored for pre-clinical trials. The tool consists of 10 items designed to assess bias risks. These items include: 1) Random sequence generation, 2) Baseline characteristics, 3) Allocation concealment, 4) Randomization of animal placement, 5) Blinding of investigators and subjects, 6) Randomized outcome assessment, 7) Blinding of outcome assessors, 8) Incomplete outcome data, 9) Selective reporting of outcomes, and 10) Other sources of bias. Each item is assigned a score of 1 point, resulting in a maximum total of 10 points. Any disagreements that arose during the risk assessment were resolved through consultation with the corresponding author.

### GRADE assessment

Following the GRADE guidelines, the certainty of evidence for primary outcomes was evaluated across five domains: risk of bias, inconsistency, indirectness, imprecision, and publication bias. Given that preclinical animal studies inherently exhibit high heterogeneity and methodological variability, the initial quality of evidence was predefined as “low” in accordance with established expert consensus for animal research meta-analyses.

### Statistical analysis of data

Using Stata 15, we calculated Standardized Mean Differences (SMD) with 95% confidence intervals (CI) for continuous data. The choice of pooling method relied on the I^2^ heterogeneity metric: a fixed-effects model was utilized for homogenous data (I^2^ < 50%), and a random-effects model was employed when significant heterogeneity (I^2^ > 50%) was detected. For primary outcome measures that exhibited significant heterogeneity, we will explore sources of heterogeneity through sensitivity analyses and subgroup analyses. Sensitivity analyses will be performed using the “db metaninf” command. Significance will be determined by a p < 0.05.

### Publication bias

For outcomes including over ten studies, publication bias was assessed via funnel plot asymmetry and Egger’s test in Stata 15 (significance level: P < 0.05). Where substantial bias risk was observed, the trim-and-fill method was utilized to correct the data and calculate an adjusted aggregate effect, ensuring the stability of our findings.

### Dose-time-response analysis

Investigating the effects of puerarin on outcomes related to NAFLD necessitates an examination of efficacy across different intervention durations and dosing regimens, thereby generating time-dose-response curves. For primary outcome measures included in over 10 studies, graphical analyses were generated to elucidate the correlations among administered dose, treatment course, and clinical efficacy.

## Results

### Search results

**FIGURE 2:**
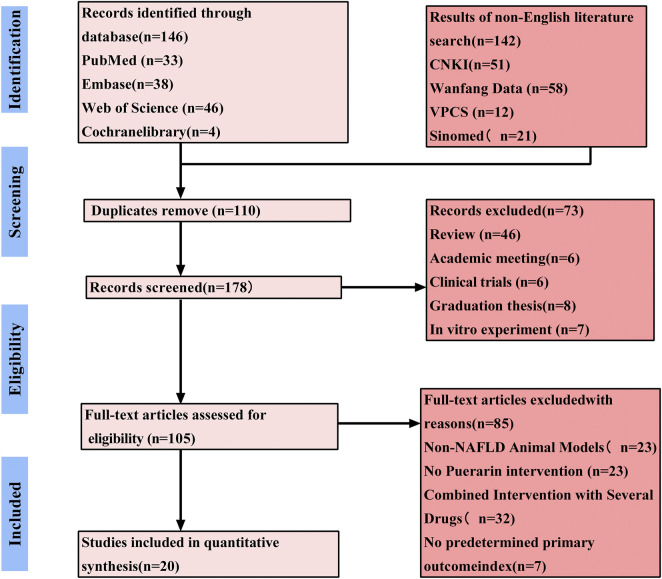
Prisma flow diagram of study selection process.

A total of 288 articles were retrieved via various databases: Embase (n = 63), Wanfang Data (n = 58), CNKI (n = 51), Web of Science (n = 46), PubMed (n = 33), Sinomed (n = 21), VPCS (n = 12), and the Cochrane Library (n = 4). Following the aggregation of search yields and the removal of duplicate records, 178 citations were retained. We performed a preliminary review of titles and abstracts, removing 73 records for the specific reasons listed below: 1) review articles, 2) conference proceedings, 3) clinical trials, 4) *in vitro* experiments, and 5) degree theses. Examination of the full text led to the rejection of another 85 records owing to: 1) utilization of models unrelated to NAFLD, 2) studies not administering puerarin, 3) use of concomitant or combined therapies, and 4) outcomes of interest were not reported. Ultimately, 20 studies satisfied all inclusion criteria. Figure 2 depicts the complete PRISMA selection and attrition process.

### Study characteristics

In total 20 studies were analyzed, encompassing 331 animal models of NAFLD. Among these, 8 studies utilized Sprague-Dawley rats (161/331, 48.64%), 5 studies employed C57BL/6J mice (68/331, 20.54%), 5 studies utilized C57BL/6 mice (68/331, 20.54%), 1 study used Wistar rats (20/331, 6.04%), and 1 study did not specify the mouse strain (14/331, 4.23%). Of the 20 studies, male subjects were utilized in 17, whereas only one employed females, and two failed to report this information. 11 studies reported animal weights, while 9 did not provide this data. Concerning the modeling approaches for NAFLD, 14 studies employed a high-fat diet (HFD), 3 used a methionine-choline-deficient diet (MCD), 1 utilized a high-fat high-fructose diet (HFFD), and 2 employed a high-fat high-sucrose diet (HFHSD), certain model characteristics highly replicate the core metabolic drivers of NAFLD observed in the current Chinese population. The dosage regimens for puerarin spanned from 100 to 800 mg/kg per day. The intervention duration ranged from 2 weeks to 18 weeks. All interventions were delivered via oral gavage. In terms of outcome measures, 12 studies assessed serum TG and TC levels in animals, while 9 studies evaluated TG and TC levels in liver tissue. Additionally, 13 studies reported ALT and AST levels, and 9 studies documented LDL-C and HDL-C. Furthermore, several studies reported inflammatory markers and antioxidant markers. The main features of the included animal studies are outlined in Table 1.

**TABLE 1:** Basic characteristics of included studies.

Study (Year)	Species (Sex, age, n = treatment/control group, Weight)	Modeling method	Treatment group (Puerarin) (purity, dose, course of treatment, administration)	Control group	Outcome	Intergroup differences
Zhang et al. (2024)	NR mice (male, 6 weeks, 7/7, NR)	HFD	Puerarin, ≥98%, 100 mg/kg/d, 16 days, by Intragastric	Equal amount of normal saline	TG (serum)	p < 0.001
					TC (serum)	p < 0.01
					HDL-C	p < 0.001
					HDL-C	p < 0.001
Ding et al. (2024)	C57BL/6J mice (male, 6 weeks, 6/6, 20 ± 2 g)	HFHSD	Puerarin, ≥98%, 400 mg/kg/d, 8 weeks, by Intragastric	Equal amount of normal saline	TNF-α	p < 0.01
					IL-6	p < 0.01
					IL-1β	p < 0.01
Hu et al. (2021)	SD rats (male, 6–8 weeks, 10/10, 180–200 g)	HFD	Puerarin, ≥98%, 100 mg/kg/d, 8 weeks, by Intragastric	Equal amount of 9.0 g/L Sodium Chloride Solution	ALT	p < 0.05
					AST	p < 0.05
					TG (serum)	p < 0.05
					TC (serum)	p < 0.05
					LDL-C	p < 0.05
					HDL-C	p < 0.05
					TNF-α	p < 0.05
					IL-6	p < 0.05
Jin et al. (2020)	SD rats (male, NR, 10/10, 180–220 g)	HFD	Puerarin, ≥98%, 250 mg/kg/d, 4 weeks, by Intragastric	Equal volume of double-distilled water	TG (serum)	p < 0.05
					TC (serum)	p < 0.05
					HDL-C	p > 0.05
					HDL-C	p < 0.05
					ALT	p < 0.05
					AST	p < 0.05
					IL-1β	p < 0.05
					TNF-α	p > 0.05
Leng et al. (2018)	SD rats (male, 6 weeks, 8/8, 180–200 g)	HFD	Puerarin, ≥98%, 250 mg/kg/d, 4 weeks, by Intragastric	Not provided	ALT	p < 0.05
					AST	p < 0.05
					TC (serum)	p < 0.05
					TG (serum)	p < 0.05
					HDL-C	p < 0.05
					LDL-C	p < 0.05
					SOD	p < 0.05
					MDA	p < 0.05
Zheng et al. (2008a)	SD rats (male, NR, 12/10, 180 ± 30 g)	HFD	Puerarin, >98%, 300 mg/kg/d, 4 weeks, by Intragastric	2 mL 0.9% Sodium Chloride Solution	TC (liver)	p < 0.01
					TG (liver)	p < 0.01
Zheng et al. (2008b)	SD rats (male, NR, 15/12, 180 ± 10 g)	HFD	Puerarin, ≥98%, 800 mg/kg/d, 4 weeks, by Intragastric	Equal amount of normal saline	TC (liver)	p < 0.05
					TG (liver)	p < 0.05
Han et al. (2026)	C57BL/6J mice (male, 6 weeks, 6/6, NR)	HFD	Puerarin, ≥98%, 300 mg/kg/d, 4 weeks, by Intragastric	Equal amount of normal saline	TG (serum)	p < 0.01
					TC (serum)	p < 0.01
					ALT	p < 0.05
					AST	p > 0.05
					MDA	p < 0.01
					SOD	p < 0.05
Tang et al. (2024)	C57BL/6J mice (male, NR, 6/6, NR	HFD	Pueraria lobata root 1950 mg/kg/d (contains 23% puerarin, approximately 448.5 mg/kg/d), 4 weeks, by Intragastric	Equal amount of normal saline	TG (serum)	p < 0.001
					TC (serum)	p < 0.001
					LDL-C	p < 0.001
					HDL-C	p < 0.001
					ALT	p < 0.001
					AST	p < 0.001
					TNF-α	p < 0.001
					IL-6	p < 0.001
Dong et al. (2024)	Wistar rats (famale,6–8 weeks, 10/10, 180–220 g)	HFD	Puerarin,NR,NR, 4 weeks, by Intragastric	Distilled water (10 mL/kg/day)	TC (serum, liver)	p < 0.01
					TG (serum, liver)	p < 0.01
					LDL-C	p < 0.01
					HDL-C	p < 0.05
					AST	p < 0.01
					ALT	p < 0.01
					SOD	p < 0.05
					MDA	p < 0.05
					GSH-Px	p < 0.01
Wang et al. (2019)	C57BL/6J mice (male, 6–8weeks, 10/10, NR)	HFHSD	Puerarin, NR, 400 mg/kg/d, 18 weeks, by Intragastric	Not provided	ALT	p < 0.05
					AST	p < 0.05
Ling et al. (2025)	SD rats (male, NR, 6/6, NR)	HFD	Puerarin, NR, 400 mg/kg/d, 7 weeks, by Intragastric	Equal amount of normal saline	TC (serum, liver)	p < 0.01
					TG (serum, liver)	p < 0.01
					LDL-C	p < 0.01
					HDL-C	p < 0.01
					AST	p < 0.01
					ALT	p < 0.01
					IL-IB	p < 0.01
					IL-6	p < 0.01
					TNFA	p < 0.01
Li Z. et al. (2024)	C57BL/6 mice (NR, 6–8 weeks, 8/8, NR)	HFD	Puerarin, NR 300 mg/kg/d, 8 weeks, by Intragastric	Not provided	TC (liver)	p < 0.01
					TG (liver)	p < 0.01
					ALT	p < 0.05
					AST	p < 0.05
					MDA	p < 0.001
					SOD	p < 0.001
					GSH-Px	p < 0.001
Yang et al. (2023)	C57BL/6J mice (male, NR, 6/6, NR)	HFD	Puerarin,NR, 200 mg/kg/d, 4 weeks, by Intragastric	Equal volume of sodium carboxymethylcellulose without puerarin	TG (serum)	p < 0.05
					TC (serum)	p < 0.01
					AST	p < 0.01
					ALT	p < 0.01
					TNF-α	—
					Il-1β	—
					Il-6	—
					GSH-Px	p < 0.001
					SOD	p < 0.001
					MDA	p < 0.05
Zhou et al. (2022)	SDrats (male, 8 weeks, 10/10, 180–220 g)	HFFD	Puerarin, ≥98%, 200 mg/kg/d, 4 weeks, by Intragastric	Not provided	TG (serum)	p < 0.01
					TG (liver)	p < 0.01
					TC (liver)	p < 0.01
					AST	p < 0.01
					ALT	p < 0.01
					SOD	p < 0.01
					GSH-Px	p < 0.05
					MDA	p < 0.01
					IL-1β	p < 0.01
					TNF-α	p < 0.05
Guo et al. (2019)	SD rats (male, 7–8 weeks, 12/12, 160–170 g)	HFD	Puerarin, NR, 800 mg/kg/d, 4 weeks, by Intragastric	Equal amount of normal saline	TC (liver)	p < 0.05
					TG (liver)	p < 0.05
Gong et al. (2021)	C57BL/6 mice (male, 8weeks, 6/6, NR)	MCD	Puerarin, ≥98%, 400 mg/kg/d, 4weeks, by Intragastric	Equal amount of normal saline	TC (serum, liver)	p > 0.05, p > 0.05
					TG (serum, liver)	p > 0.05, p < 0.01
					ALT	p < 0.01
					AST	p < 0.05
Wang et al. (2014)	C57BL/6 mice (NR, NR, 8/8, 20–25 g)	MCD	MCD + Puerarin 0.1 mL/10 g, NR, 2 weeks, by Intragastric	Not provided	TG (serum)	p > 0.05
					TC (serum)	p > 0.05
					HDL-C	p > 0.05
					LDL-C	p < 0.01
					TNF-α	p < 0.01
					IL-6	p > 0.05
Zhong et al. (2023)	C57BL/6 mice (male, 6–8 weeks, 6/6, 20–25 g)	MCD	MCD diet containing 0.5% w/w puerarin, NR, NR, by Intragastric	Not provided	TC	p < 0.05
					TG	p < 0.01
					ALT	p < 0.01
					AST	p < 0.05
					SOD	p < 0.05
					GSH-Px	p < 0.05
					MDA	p < 0.01
					TNF-α	p < 0.01
					Il-1β	p < 0.01
Sun et al. (2025)	C57BL/6 mice (male, 6weeks, 6/6, NR)	HFD	Puerarin, NR, 200 mg/kg/d, 4 weeks, by Intragastric	Equal amounts of saline at a dose of 0.2 mL	TC (serum)	p < 0.01
					LDL-C	p < 0.001
					HDL-C	p < 0.01
					TNF-α	p < 0.01
					Il-1β	p < 0.01
					Il-6	p < 0.01

TC, Total Cholesterol; TG, Triglyceride; LDL-C, Low-Density Lipoprotein Cholesterol; HDL-C, High-Density Lipoprotein Cholesterol; ALT, Alanine Aminotransferase; AST, Aspartate Aminotransferase; IL-6, Interleukin-6; IL-1β, Interleukin-1 Beta; TNF-α, Tumor Necrosis Factor Alpha; SOD, Superoxide Dismutase; MDA, Malondialdehyde; GSH-Px, Glutathione Peroxidase; MCD, Methionine-Choline-Deficient Die; HFD, High-Fat Diet; HFHSD, High-Fat High-Sucrose Diet; HFFD, High-Fat Fructose Diet. NR, Not Reported.

### Quality assessment

Assessment of methodological rigor across the 20 eligible studies yielded scores spanning from 5 to 7. Three studies scored 5 points, seven scored 6 points, and ten achieved the maximum score of 7 points. All studies reported the use of randomization; however, none provided specific details regarding the randomization method employed. Baseline characteristics were not reported in two studies. Animal housing conditions were described in 14 studies but omitted in the remaining six. Three studies failed to incorporate every animal subject into the concluding data evaluation. The reported outcomes of all 20 studies were consistent with their respective experimental protocols. Zero studies disclosed allocation secrecy, blinding protocols, or alternative sources of bias. Further details are presented in Supplementary Figures 1, 2.

### GRADE assessment results

The certainty of evidence for the primary outcome measures, including TG, TC, ALT, and AST, was assessed using the GRADE framework. The comprehensive results of this assessment are summarized in Table 2.

**TABLE 2:** GRADE evidence profile for the effects of puerarin on NAFLD.

Outcomes	Number of animals	Initial Quality	Risk of bias	Inconsistency	Indirectness	Imprecision	Publication bias	Final certainty level
Serum TG	12 (186)	High	Serious (−1)^a^	Serious (−1)^b^	Not serious	Not serious	Serious (−1)^c^	Very Low
Serum TC	12 (178)	High	Serious (−1)^a^	Serious (−1)^b^	Not serious	Not serious	Serious (−1)^c^	Very Low
ALT	13 (204)	High	Serious (−1)^a^	Serious (−1)^b^	Not serious	Not serious	Serious (−1)^c^	Very Low
AST	13 (204)	High	Serious (−1)^a^	Serious (−1)^b^	Not serious	Not serious	Serious (−1)^c^	Very Low

^a^ Risk of Bias: All 20 included studies failed to report specific details regarding allocation concealment and blinding. Based on the SYRCLE tool assessment, the certainty of evidence was downgraded by 1 level.

^b^ Inconsistency: Significant statistical heterogeneity was observed across all outcomes (I2 ranged from 78.2% to 91.2%, p < 0.01). Furthermore, results were significantly influenced by animal strains and modeling methods; therefore, the certainty was downgraded by 1 level.

^c^ Publication Bias: Egger’s test indicated significant publication bias for TG, TC, ALT, and AST (p < 0.05$). Consequently, the certainty was downgraded by 1 level.

### Preclinical effects of puerarin on NAFLD models

#### Primary outcomes

##### Lipid metabolism reactions

**FIGURE 3:**
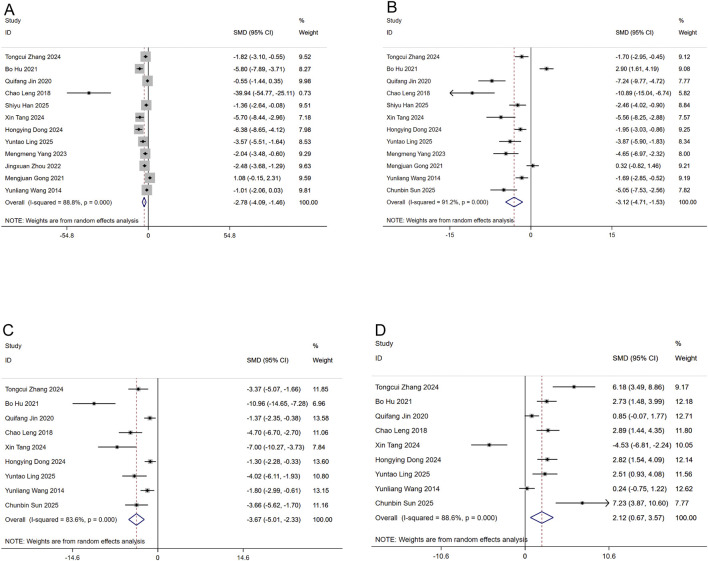
Forest plots for lipid metabolic reactions to puerarin treatment **(A)** TG **(B)** TC **(C)** LDL-C **(D)** HDL-C.

Aggregated analyses of twelve investigations demonstrated that puerarin administration significantly suppressed both serum TG (n = 186) and TC levels (n = 178) relative to control groups, with SMDs of −2.78 (95% CI: −4.09, −1.46) and −3.12 (95% CI: −4.71, −1.53), respectively (Figures 3A,B). In parallel, data from nine studies (n = 142) indicated that puerarin was effective in optimizing lipoprotein profiles; it significantly reduced LDL-C (SMD: −3.67; 95% CI: −5.01, −2.3; Figure 3C) and concurrently elevated HDL-C levels (SMD: 2.12; 95% CI: 0.67, 3.57; Figure 3D) compared to controls. All analyses showed high heterogeneity (I^2^ ranges: 83.6%–91.2%, P < 0.01).

##### Lipid accumulation in the liver

**FIGURE 4:**
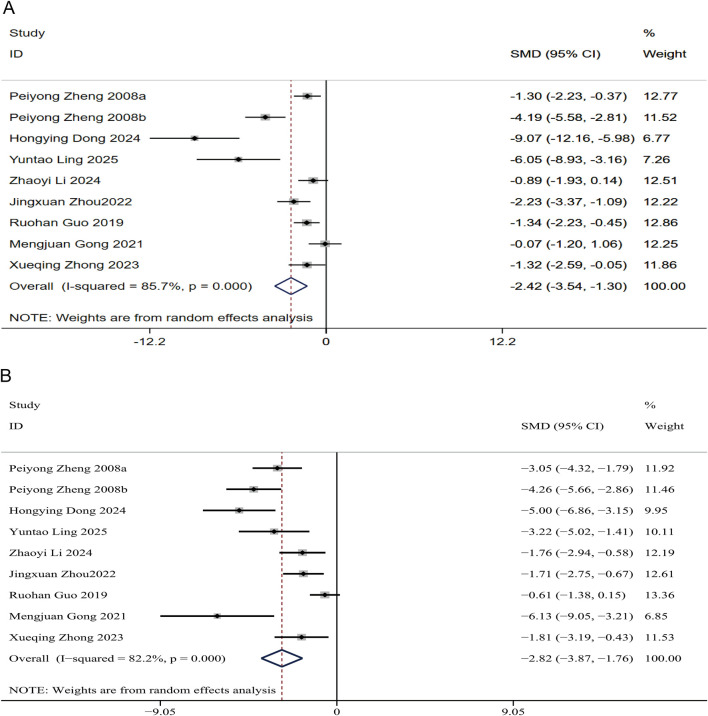
Forest plots for hepatic lipid accumulation indicators in response to puerarin treatment **(A)** Hepatic TC **(B)** Hepatic TG.

A comprehensive analysis of NAFLD indicates that lipid accumulation in the liver is the primary mechanism involved. The nine studies included in this analysis demonstrate a marked reduction in TG and TC in the livers of the treatment groups. Notably, among the 165 NAFLD animal models examined, the combined SMD for liver TG was −2.82 (95% CI: −3.86, −1.77), with substantial heterogeneity observed (I^2^ = 81.4%, P < 0.01) (Figure 4A). Similarly, the combined SMD for liver TC was −2.42 (95% CI: −3.54, −1.30), also exhibiting high heterogeneity (I^2^ = 85.7%, P < 0.01) (Figure 4B). These findings suggest that puerarin may effectively reduce fat deposition in liver cells.

##### Liver function indicators

**FIGURE 5:**
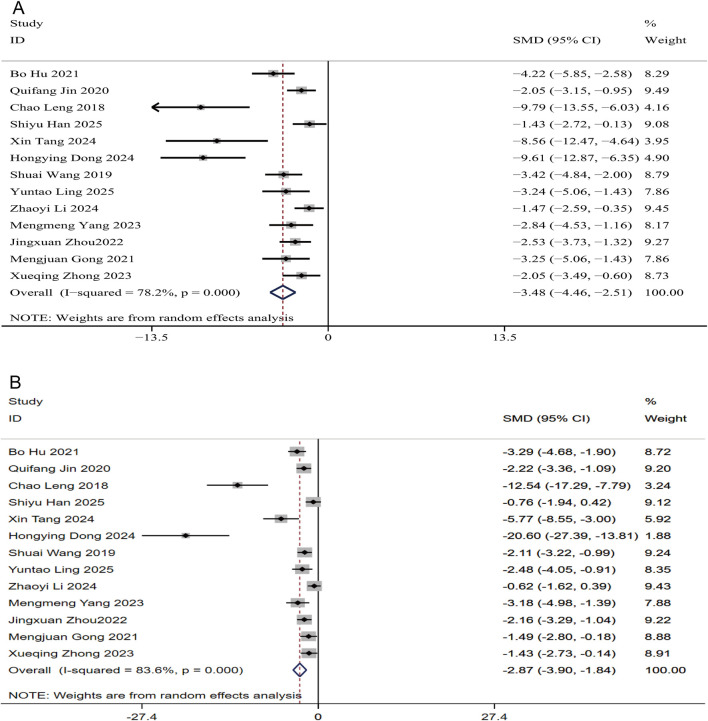
Forest plots for liver function indicators in response to puerarin treatment **(A)** ALT **(B)** AST.

In the assessment of liver injury, ALT and AST serve as critical indicators of hepatocellular damage. The puerarin group exhibited a significant reduction in both ALT and AST levels compared to the control group. A total of 204 NAFLD animal models were analyzed across 13 studies. The SMD for ALT was −3.48 (95% CI: −4.46, −2.51), heterogeneity: I^2^ = 78.2%, P < 0.01 (Figure 5A). For AST, the SMD was −2.87 (95% CI: −3.90, −1.84), heterogeneity: I^2^ = 83.6%, P < 0.01 (Figure 5B). These findings indicate that puerarin effectively reduces serum levels of ALT and AST in NAFLD rats, mitigates liver function impairment, improves pathological alterations in liver tissue structure, and provides a significant protective effect on liver function.

### Secondary outcomes

#### Inflammatory markers

**FIGURE 6:**
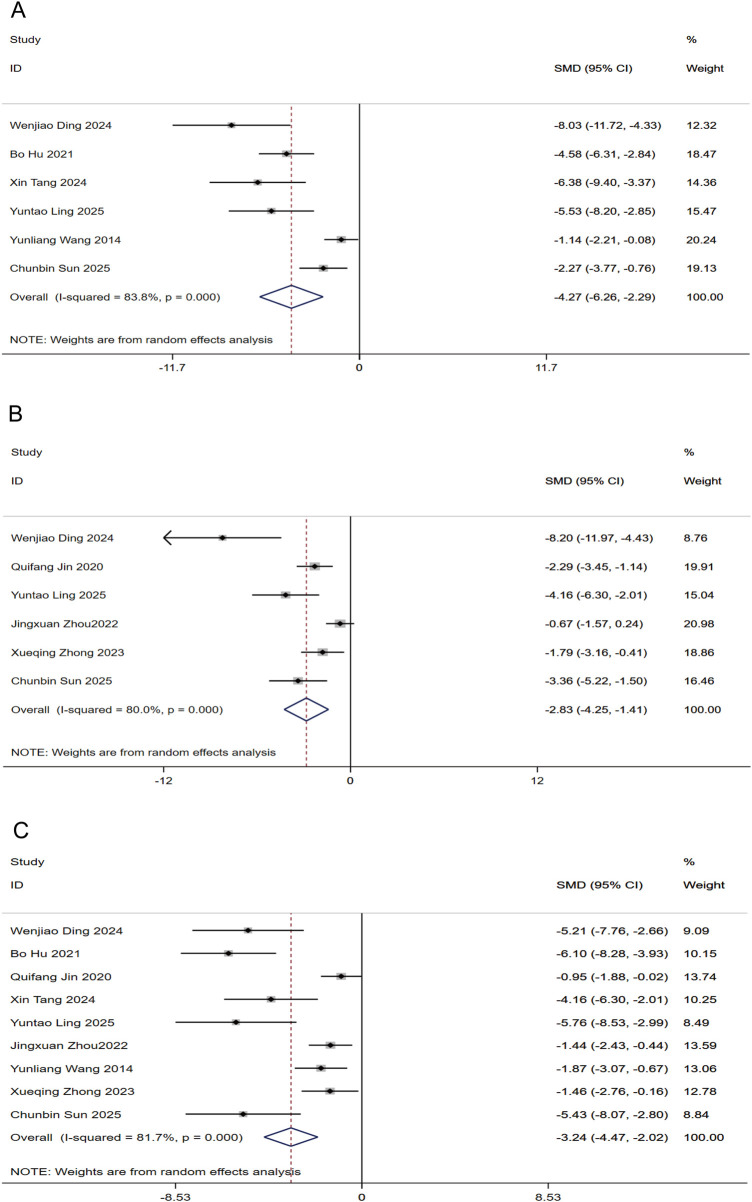
Forest plots for Inflammatory markers in response to puerarin treatment **(A)** IL-6 **(B)** IL-1β **(C)** TNF-α.

Aggregated assessments of inflammatory mediators indicated that puerarin administration led to a substantial suppression of IL-6 (six studies, n = 84) and IL-1β (six studies, n = 88), with SMDs of −4.27 (95% CI: −6.26, −2.29) and −2.83 (95% CI: −4.25, −1.41), respectively (Figures 6A,B). Furthermore, an aggregate of nine investigations confirmed a marked reduction in TNF-α levels relative to controls [n = 136, SMD: −3.24 (95% CI: −4.47, −2.02); Figure 6C]. All analyses exhibited significant statistical heterogeneity (P < 0.01).

#### Oxidative stress markers

**FIGURE 7:**
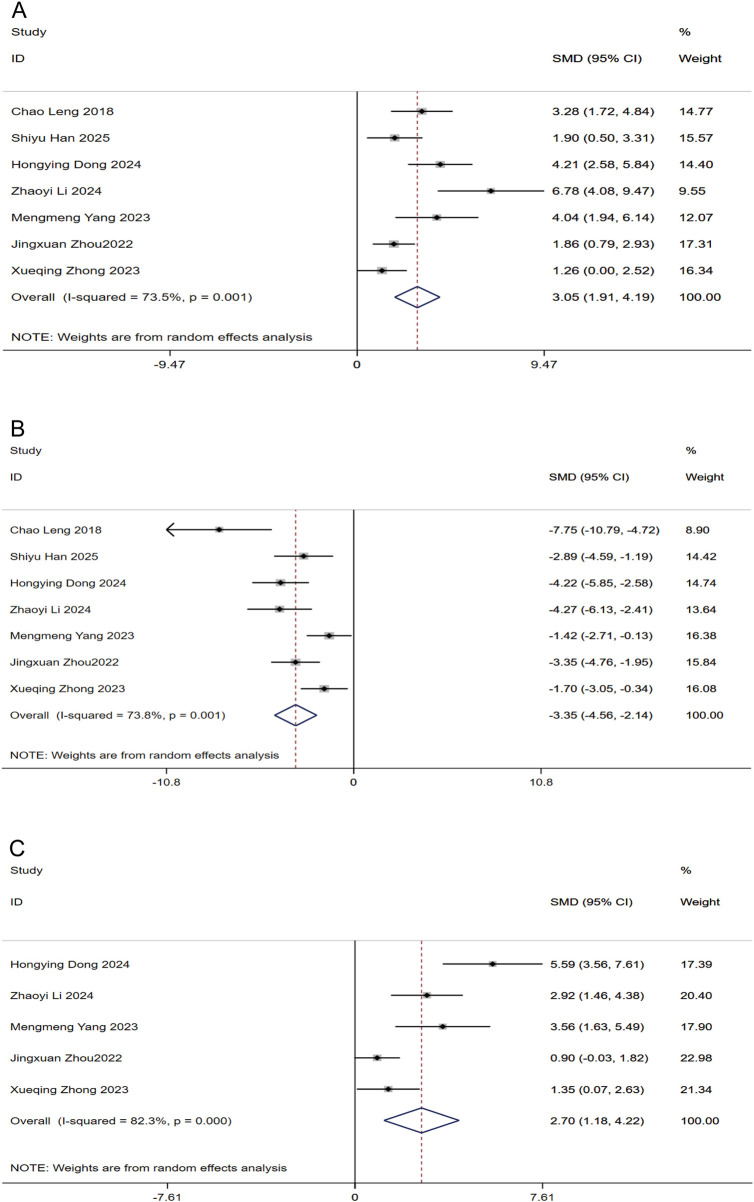
Forest plots for Oxidative stress markers in response to puerarin treatment **(A)** SOD **(B)** MDA **(C)** GSH-Px.

As shown in Figure 7, the meta-analysis revealed favorable changes in oxidative stress indices following puerarin treatment. Compared to the control groups, the intervention resulted in significantly higher SOD activity [SMD: 3.05 (95% CI: 1.91, 4.19); Figure 7A] and GSH-Px concentrations [SMD: 2.70 (95% CI: 1.18, 4.22); Figure 9C]. In parallel, a significant reduction was noted in MDA levels [SMD: −3.35 (95% CI: −4.56, −2.14); Figure 7B]. All outcomes were statistically significant (P < 0.05) but exhibited substantial heterogeneity (I^2^ ranges: 73.5%–82.3%).

### Results of sensitivity analysis

For the primary outcome measures, we determined the influence of single studies on the pooled effect size by iteratively excluding one study at a time. The results of the analysis indicate that all primary outcome measures demonstrate robust stability (Supplementary Figures 3–10).

### Subgroup analysis results

To investigate the sources of high heterogeneity among primary outcomes, subgroup analyses were conducted based on animal species, modeling methods, intervention duration, and dosage (Supplementary Figures 9–40). The results identified intervention duration as the core regulator of inter-study variance in serum and hepatic lipid profiles—specifically serum TG, liver TG, serum TC, LDL-C, and HDL-C—suggesting that the remodeling of lipid metabolism by Puerarin is significantly time-dependent. Furthermore, the high heterogeneity observed in liver TC, LDL-C, and ALT was closely associated with animal strains (genetic background) and modeling approaches (pathological triggers). In contrast, the variance in AST, a marker of deep hepatocellular injury, was highly dependent on specific modeling methods, such as the severe necrosis induced by a MCD diet. Detailed factors contributing to the high heterogeneity of primary outcomes are summarized in Table 3.

**TABLE 3:** Analysis of heterogeneity sources.

Primary source of heterogeneity	Significantly affected outcome measures
Intervention duration	Serum TG, liver TG, serum TC and LDL-C, HDL-C
Animal species	Liver TC, LDL-C and ALT
Modeling method	Liver TC, LDL-C, ALT and AST

### Publish biased results

**FIGURE 8:**
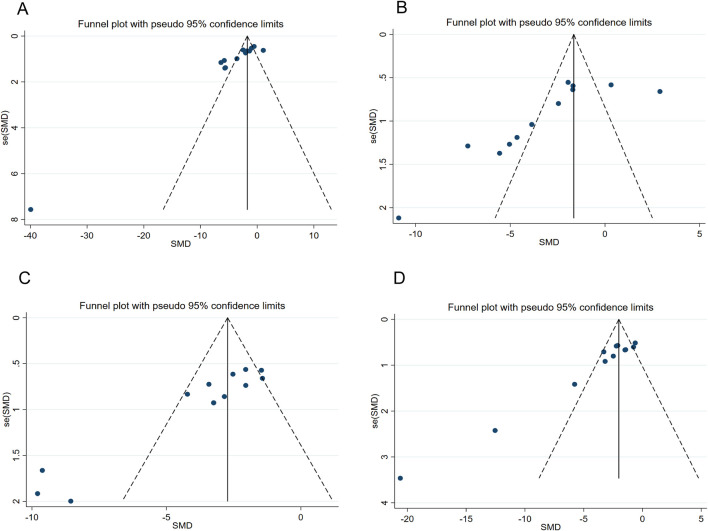
Funnel plot for **(A)** TG **(B)** TC **(C)** ALT **(D)** AST.

**FIGURE 9:**
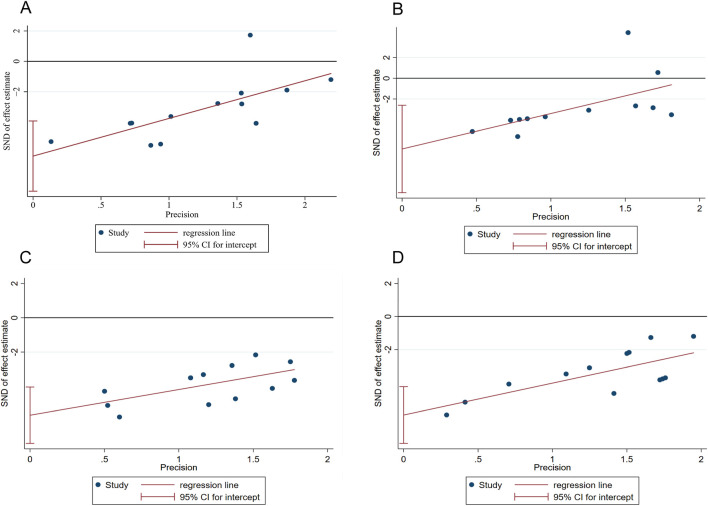
Eggerʼs publication bias plot for **(A)** TG **(B)** TC **(C)** ALT **(D)** AST.

**FIGURE 10:**
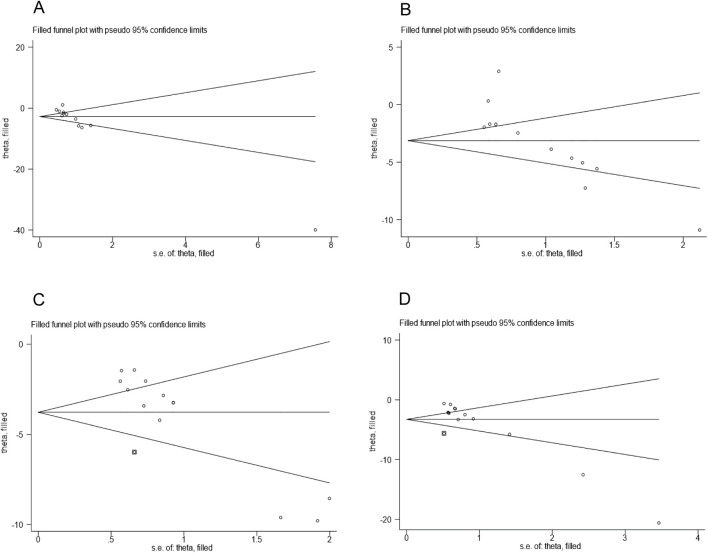
Trim-and-fill analysis for **(A)** TG **(B)** TC **(C)** ALT **(D)** AST.

Publication bias assessment was performed for outcome measures (TG, TC, ALT, AST) that included more than 10 studies. The points in the funnel plot exhibited an asymmetrical distribution (Figure 8). Additionally, the results of Egger’s test (Figure 9) indicated the presence of publication bias in these four outcome measures (P < 0.05). Consequently, we implemented adjustments using data pruning and padding methods, which did not significantly alter the significance of the effect value. The results demonstrated good robustness (Figure 10), suggesting that publication bias does not have a substantial impact on the findings of the meta-analysis.

### Dose-time-response visualization

**FIGURE 11:**
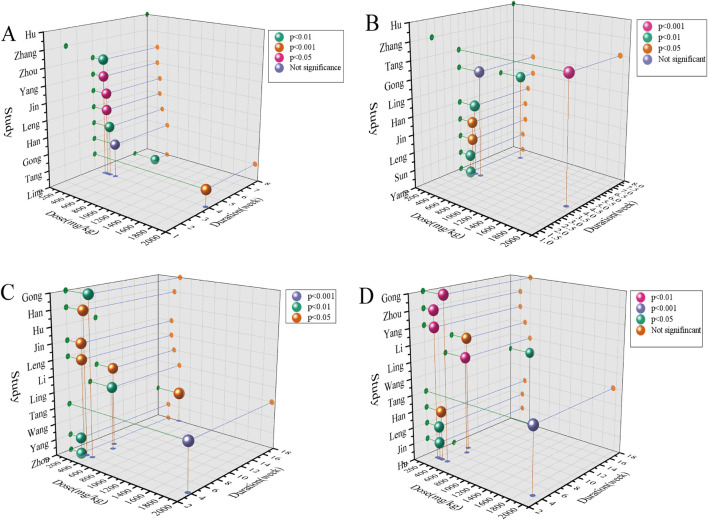
Dose-time-response diagrams of puerarin on **(A)** TG **(B)** TC **(C)** ALT and **(D)** AST. Each sphere represents the improvement rate of the treated subgroups with statistically significant differences (P < 0.05). The x-axis indicates the intervention duration (week), and the y-axis indicates the dose (mg/kg).

In this study, we integrated the dosage and treatment cycle of the puerarin intervention group with the effect results of TG, TC, AST, and ALT indicators from the included studies (P < 0.05) to establish a time-dose association protocol. This protocol provides a valuable reference for subsequent related research. Puerarin exhibited marked hypolipidemic activity, significantly suppressing TG and TC levels when administered at dosages ranging from 100 to 400 mg/kg over a period of 16 days to 8 weeks. Furthermore, this same dosage spectrum was associated with a downward trajectory in hepatic enzymes (AST and ALT) across intervention durations lasting between 4 and 18 weeks. One of the included studies examined kudzu root extract, which contains 23% puerarin, resulting in a relatively high dosage. The deviation illustrated in the Figureure was notably significant; therefore, this study was excluded as a reference. In summary, the current data demonstrate that puerarin possesses marked efficacy in regulating lipid metabolism and preserving hepatic function, with therapeutic outcomes that are contingent upon both dosage and treatment duration (Figure 11). Each sphere in the figure represents the improvement rate of a treatment subgroup with statistical significance. The x-axis indicates the duration of intervention (week), the y-axis represents the dose (mg/kg), and the z-axis denotes the included studies.

## Discussion

### Summary of evidence

This study encompassed 20 preclinical trials involving a total of 331 experimental animals, confirming that puerarin exhibits protective efficacy against animal models of NAFLD. Through an integrated analysis of the research data, we determined that puerarin not only reduces levels of lipid metabolism-related indicators, but also markedly protects liver function. In addition, beneficial changes were observed in mechanism-associated variables; specifically, puerarin optimized the levels of inflammatory markers and oxidative stress metrics. Current evidence implies that the therapeutic efficacy of puerarin against NAFLD is likely mediated by a synergistic mechanism involving the restoration of lipid homeostasis, suppression of inflammation, and mitigation of oxidative stress. Nevertheless, significant statistical heterogeneity characterized the pooled results for all primary endpoints, including TG, TC, LDL-C, HDL-C, AST, and ALT. To elucidate the origins of this variability, we conducted stratified analyses, which suggested that the observed inconsistency was likely attributable to differences in disease induction models, animal species, dosage regimens, and the length of treatment. Furthermore, for outcome measures (such as TG, TC, ALT, and AST) with more than 10 included studies, we conducted evaluations for publication bias and employed pruning and padding methods to supplement potentially missing studies. The adjusted data showed no significant changes, indicating that the included studies were stable and reliable.

### Analysis of mechanisms

Based on a comprehensive review of the eligible studies, this paper outlines the putative mechanisms underlying the hepatoprotective efficacy of puerarin in NAFLD animal models.

### Regulate lipid metabolism

Lipid metabolism disorder constitutes the primary pathological mechanism underlying NAFLD, with hepatic steatosis as its immediate consequence. This condition can advance to severe complications, including hepatitis and liver fibrosis. Consequently, the regulation of lipid metabolism disorders is essential for the comprehensive prevention and treatment of NAFLD (Dai et al., 2025). These disorders are associated with an imbalance in *de novo* lipogenesis (DNL) and fatty acid β-oxidation (FAO).

DNL serves as a critical pathogenic link to lipid metabolism disorders, with its activity meticulously regulated by factors such as sterol regulatory element-binding protein 1c (SREBP-1c), fatty acid synthase (FAS), and stearoyl-CoA desaturase 1 (SCD-1) (Batchuluun et al., 2022). Notably, the aberrantly elevated expression of SREBP-1c protein enhances the *de novo* synthesis of fatty acids in the liver, exacerbating hepatocyte steatosis (Ferré et al., 2021). Research indicates that puerarin significantly decreases the mRNA expression levels of SREBP-1c in the livers of mice subjected to a HFD model. By inhibiting the transcriptional activity of SREBP-1c, puerarin reduces the expression of liver lipid synthesis genes, consequently lowering serum TC and LDL-C levels. This inhibition of fat synthesis further mitigates the accumulation of lipid droplets in the liver (Sun et al., 2025).

**FIGURE 12:**
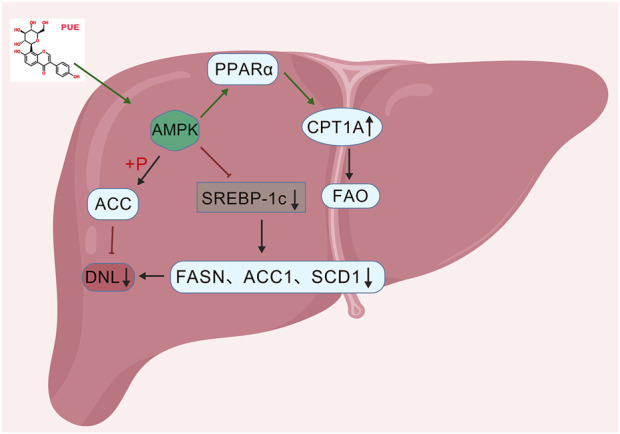
Mechanism of puerarin in regulating lipid metabolism.

As a critical and highly conserved modulator of energy balance, Adenosine 5′-monophosphate-activated protein kinase (AMPK) remains a central target in research concerning metabolic disorders (Yadav et al., 2025). AMPK regulates adipogenesis primarily *via* the phosphorylation of its critical downstream target, acetyl-CoA carboxylase (ACC). This modification results in a marked decrease in fatty acid production and the concurrent suppression of enzymes involved in DNL (Fang et al., 2022). Research indicates that puerarin can activate AMPK, resulting in the inhibition of downstream activity of SREBP-1c and a subsequent downregulation of the expression levels of ACC1, fatty acid synthase (FASN), and stearoyl-CoA desaturase 1 (SCD1) (Xu et al., 2021). This process effectively inhibits the *de novo* synthesis of fat and cholesterol. Hepatic AMPK signaling upregulates lipid catabolism primarily through the promotion of FAO. FAO is governed by a regulatory network involving carnitine palmitoyltransferase 1A (CPT1A) and peroxisome proliferator-activated receptor α (PPARα) (Schlaepfer et al., 2020). CPT1A functions as the rate-limiting enzyme that facilitates the entry of fatty acids into mitochondria for β-oxidation. An increase in CPT1A expression can promote the transport of free fatty acids (FFA) to the mitochondrial matrix (He et al., 2025). As a nuclear receptor, PPAR-α regulates the transcription of genes associated with fatty acid oxidation, including CPT1A, thereby enhancing mitochondrial β-oxidation capacity (Higa et al., 2025). Puerarin has been shown to improve NAFLD outcomes by triggering the PPAR-α cascade and increasing CPT1A levels, thereby facilitating the breakdown of fatty acids *via* mitochondrial β-oxidation (Zhou et al., 2022). By identifying FAO as a primary therapeutic target (Figure 12), this study elucidates the molecular basis of puerarin’s efficacy, reinforcing the rationale for targeted interventions in metabolism-associated liver diseases.

### Modulating the gut microbiota-metabolism axis

Abnormal hepatic metabolism contributes to the promotion of fatty liver disease, while dysbiosis of the gut microbiota further exacerbates NAFLD. The underlying mechanisms primarily involve the impairment of tight junction integrity and intestinal hyperpermeability, infiltration of lipopolysaccharides (LPS) and inflammatory factors, reduced production of short-chain fatty acids (SCFAs), and enhanced ethanol biosynthesis (Wang H. et al., 2025; Zhang et al., 2025b). When LPS breaches the bloodstream through a compromised intestinal barrier, it initiates systemic inflammatory responses, particularly intensifying hepatic inflammation and steatosis (Chen L. et al., 2025). SCFAs, which are primary metabolites generated by gut microbiota during the fermentation of dietary fiber, include acetate, propionate, and butyrate (Shashni et al., 2023). These metabolites upregulate the expression of intestinal tight junction proteins (ZO-1, Occludin, Claudin-1), diminish intestinal permeability, and inhibit the entry of pro-inflammatory substances such as LPS into the bloodstream (Cheng et al., 2020; Ran et al., 2022).

Dysbiosis is marked by an increased Firmicutes/Bacteroidetes ratio and abnormal levels of SCFAs (Wang et al., 2020). A study by Sun Chunbin’s group highlights that puerarin restores microbial balance by increasing the abundance of Bacteroidetes and reducing the Firmicutes/Bacteroidetes proportion. Additionally, the compound was shown to augment the prevalence of SCFA-producing genera, including Muribaculum and Dubosiella. This alteration in microbial composition elevates intestinal levels of acetate, propionate, and butyrate. Furthermore, it reshapes the microbial structure, upregulates the expression of tight junction proteins, decreases LPS translocation into the bloodstream, and repairs the intestinal barrier. Additionally, puerarin indirectly inhibits SREBP-1c, activates essential cholesterol metabolism genes (HMG-CoA, ABCG5/8), promotes lipid breakdown and excretion, and further enhances hepatic lipid metabolism (Sun et al., 2025).

Research involving MCD diet-induced NASH models demonstrates a marked elevation in *Helicobacter* abundance within the gut microbiota of these subjects. This bacterial genus synthesizes LPS, which initiate hepatic inflammation and worsen lipid accumulation through the gut-liver axis (Schneider et al., 2019). Treatment with puerarin mitigates hepatic lipid accumulation and the inflammatory response by inhibiting LPS-producing strains, such as *Helicobacter*, while promoting SCFA-producing strains like Roseburia. This intervention restructures the gut microbiota, enhances intestinal barrier function, and decreases the release of inflammatory factors (Gong et al., 2021). In animal models of NAFLD induced by a high-fat diet, the α-diversity of gut microbiota is significantly reduced, as reflected by decreased Shannon and Chao1 indices (Yang et al., 2025). Studies have shown that puerarin intervention can effectively reverse this gut dysbiosis. The underlying mechanism involves puerarin reducing the abundance of pro-inflammatory genera such as Ruminococcus while increasing the abundance of beneficial genera such as Subdoligranulum, thereby restoring microbial diversity. This increase in beneficial genera like Subdoligranulum may improve host metabolism by promoting lipid oxidation and suppressing lipogenesis. These metabolic improvements are evidenced by reduced levels of TG, TC, and LDL-C in both liver and serum, elevated HDL-C levels, and attenuated hepatic steatosis (Ling et al., 2025).

**FIGURE 13:**
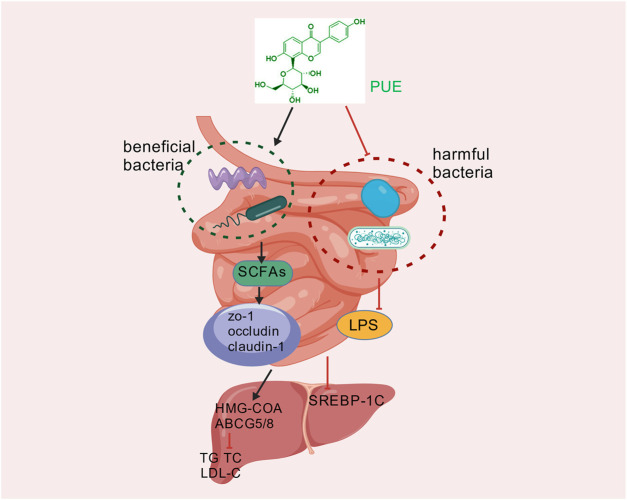
Mechanism of puerarin in regulating the gut microbiota-metabolism axis.

In summary, puerarin inhibits hepatic lipid synthesis, enhances lipid catabolism and excretion, and ultimately ameliorates hepatic lipid accumulation and related pathological conditions through multifaceted mechanisms. This process involves the restoration of α-diversity in the gut microbiota, the regulation of specific functional bacterial genera, the elevation of SCFA levels, and the repair of the intestinal barrier via signaling pathways mediated by the gut microbiota-metabolism axis (Figure 13). Future investigations should focus on key molecular pathways involved in microbiota-host interactions to develop targeted formulations for specific functional bacteria and SCFAs. When combined with personalized microbiota intervention techniques, this strategy could offer a more precise, gut-targeted approach for regulating lipid metabolism in NAFLD.

### Anti-inflammatory effects

Inflammation serves as the body’s defensive response to injury, primarily involving the activation of pattern recognition receptors by various injury factors. This activation triggers inflammatory signaling pathways, leading immune cells to release pro-inflammatory mediators and chemotactic factors (Lee and Friedman, 2022). Collectively, these mediators induce local vasodilation, enhance vascular permeability, and facilitate leukocyte recruitment, ultimately promoting tissue repair by eliminating injury factors (Luo et al., 2025). An increasing body of research suggests that puerarin possesses anti-inflammatory effects, particularly within the liver (Fang et al., 2024; Chen et al., 2026).

Nuclear factor erythroid 2-related factor 2 (Nrf2) serves as a critical transcription factor with dual antioxidant and anti-inflammatory roles. It mitigates inflammation by scavenging reactive oxygen species (ROS) and simultaneously suppressing NF-κB signaling to curb cytokine release (Chen et al., 2025a). Under basal conditions, Kelch-like ECH-associated protein 1 (Keap1) acts as a negative regulator, targeting Nrf2 for proteasomal degradation (Zhou et al., 2025). However, oxidative stress induces structural changes in Keap1 that stabilize Nrf2, allowing its nuclear translocation and subsequent upregulation of cytoprotective genes, including HO-1 and NQO1 (Alshamari et al., 2025). Intervention studies in NAFLD mice induced by a high-fat diet demonstrate that puerarin’s anti-inflammatory mechanism is closely associated with the Keap1/Nrf2/HO-1 pathway. Puerarin downregulates the expression of the negative regulator Keap1, promotes Nrf2 nuclear translocation, and upregulates the expression of its downstream genes HO-1 and NQO1. Additionally, puerarin enhances the activity of SOD and GSH-Px to scavenge ROS, while reducing the production of the lipid peroxidation byproduct MDA. This cascade inhibits ROS-mediated inflammatory pathways, lowers pro-inflammatory factors in liver tissue, and alleviates hepatic inflammatory infiltration (Chen et al., 2026).

**FIGURE 14:**
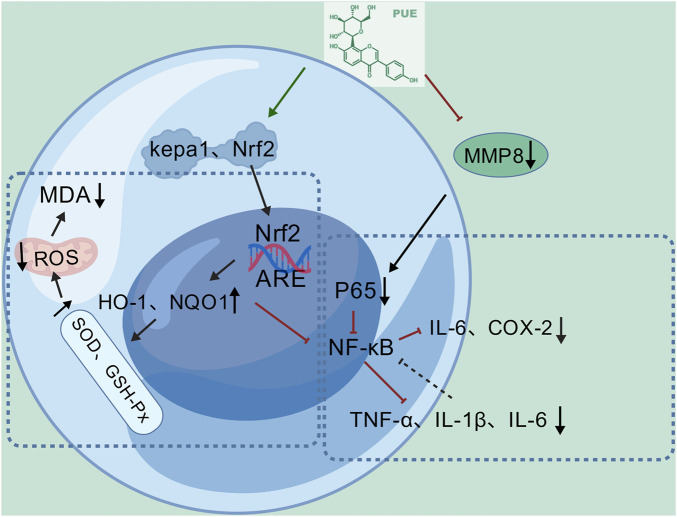
Anti-inflammatory mechanism of puerarin.

The NF-κB pathway is widely recognized as a pivotal controller of inflammatory mechanisms. This signaling trajectory initiates chiefly in response to specific upstream stimuli, including the engagement of antigen receptors, the presence of proinflammatory cytokines, and detection of pathogen-associated molecular patterns (PAMPs) (Mussbacher et al., 2023). Following activation, the NF-κB pathway induces the production of proinflammatory cytokines such as TNF-α, IL-1β, and IL-6. These cytokines can further activate NF-κB, establishing a positive feedback loop that enhances inflammatory signaling. Additionally, the pathway stimulates the release of chemokines like IL-8 and MCP-1, which drive the migration of neutrophils and monocytes/macrophages to inflamed or infected tissues (Chu et al., 2025). In a study examining the suppression of inflammation in alcoholic liver disease (Hu et al., 2023), it was demonstrated that puerarin mitigates inflammatory responses by inhibiting MMP8 activity, impeding NF-κB signaling, and decreasing the release of pro-inflammatory factors. Research shows that ethanol (EtOH)-induced alcoholic liver disease (ALD) significantly increases MMP8 activity (Forough, 2002). Functioning as a key pro-inflammatory mediator, MMP8 drives the inflammatory cascade by remodeling the extracellular matrix and modulating the recruitment of immune cells. (Ma et al., 2025). Following treatment with puerarin, MMP8 activity was markedly reduced, and the mRNA and protein levels of downstream pro-inflammatory factors, including TNF-α, IL-6, and IL-1β, were significantly downregulated (Hu et al., 2023). The NF-κB pathway, a central inflammatory signaling pathway activated by TNF-α and IL-1β, upregulates IL-6 and COX-2, thereby sustaining the inflammatory microenvironment (Liu et al., 2022). Research indicates that the MMP8 inhibitor (M8I) suppresses P65 nuclear translocation, while puerarin dose-dependently decreased p-P65 levels in liver tissue. Treatment with puerarin reversed P65 nuclear translocation without additive effects when combined with M8I, suggesting that puerarin indirectly inhibits NF-κB activation by targeting MMP8, thereby alleviating inflammatory responses in alcoholic liver disease (Figure 14) (Hu et al., 2023).

PARP-1 functions as a sensor for DNA damage. Upon cellular stress, PARP-1 is activated and acts as a co-activator of NF-κB, thereby facilitating the binding of NF-κB to DNA. This interaction significantly enhances the transcription and secretion of critical pro-inflammatory cytokines, including TNF-α, IL-1β, and IL-6 (Jin et al., 2025; Vuong et al., 2015). Additionally, activated PARP-1 can influence the PI3K/AKT signaling pathway (Xu et al., 2024; Mao et al., 2024). The PI3K/AKT pathway plays a crucial role in regulating cell survival, proliferation, growth, metabolism, and motility. The activation of AKT promotes cell activation, migration, generation of inflammatory products, potentially exacerbating inflammation through the stimulation of downstream pathways, notably NF-κB (Li et al., 2022). Studies have demonstrated (Wang et al., 2019) that puerarin ameliorates NAFLD induced by high-fat and high-sugar diets through several mechanisms. These include the inhibition of PARP-1 to reduce inflammation, the activation of the PI3K/AKT pathway, and the regulation of key lipid metabolism genes, which involves inhibiting synthesis and promoting oxidation. Additionally, puerarin enhances mitochondrial function and cellular energy, ultimately alleviating lipid accumulation, oxidative stress, and inflammatory responses.

### Antioxidant effects

Oxidative stress, a critical pathophysiological mechanism, arises from an imbalance between the production of pro-oxidants, including oxygen free radicals, and the scavenging capacity of the antioxidant defense system (Munakarmi et al., 2024). This defense system primarily comprises antioxidant enzymes such as SOD, GSH-Px, and catalase (CAT), along with non-enzymatic antioxidants like glutathione and vitamins (Pisoschi and Pop, 2015). When the accumulation of oxidants and oxygen-free radicals surpasses the clearance threshold, it leads to oxidative modifications and structural damage to macromolecules, including proteins, lipids, and DNA, thereby disrupting cellular homeostasis (Krishnamurthy et al., 2024). The liver, as the pivotal regulator of metabolism and biotransformation, exhibits heightened sensitivity to oxidative stress due to its elevated metabolic activity and complex physiological functions. This sensitivity is evidenced by the excessive activation of pro-reactive oxygen species (ROS)-generating enzymes, such as NADPH oxidase and xanthine oxidase, while mitochondrial dysfunction within hepatocytes further exacerbates the production of ROS (Allameh et al., 2023). Excessive ROS not only directly induces lipid peroxidation, generating harmful byproducts such as MDA, but also causes DNA strand breaks and collapse of the mitochondrial membrane potential. These events activate inflammatory signaling pathways, such as NF-κB, leading to persistent inflammatory responses that promote hepatic stellate cell activation and the progression of liver fibrosis, ultimately resulting in multidimensional damage to liver tissue (Wu et al., 2024). Therefore, pharmacological control of excessive oxidative stress represents a crucial strategy for preventing hepatic steatosis.

Nrf2 functions as a crucial regulatory hub in cellular responses to oxidative stress (Cheng et al., 2024). In conditions of oxidative stress induced by high glucose, reactive ROS accumulate significantly within cells. At this stage, Nrf2, as a principal antioxidant transcription factor, is released from the cytoplasm and translocates to the nucleus. There, it binds specifically to the antioxidant response element (ARE), activating the transcription of target genes such as heme oxygenase-1 (HO-1), quinone oxidoreductase-1 (NQO1), and glutathione peroxidase (GSH-Px), thereby initiating cellular antioxidant defense mechanisms to eliminate excess ROS (Li N. et al., 2024). Importantly, Nrf2 activation is closely linked to the AMPK pathway. During episodes of cellular energy deficiency, such as those triggered by hyperglycemic metabolic disorders, AMPK is rapidly activated. This activation not only restores metabolic homeostasis by inhibiting lipid synthesis pathways and promoting lipid oxidation pathways but also enhances antioxidant capacity through the regulation of the Nrf2 pathway (Meng et al., 2024; Lee et al., 2022). Under physiological conditions, Nrf2 forms a complex with Keap1, remaining in a silenced state subject to continuous ubiquitination and degradation. However, activated AMPK phosphorylates Keap1, inducing a conformational change that diminishes Keap1’s binding and inhibitory effects on Nrf2 (Qiu et al., 2020).

Extensive research demonstrates that Nrf2 is crucial for augmenting the body’s antioxidant capacity through the regulation of antioxidant enzymes, including SOD, GSH-Px, and CAT (Chen et al., 2025b; Jiang et al., 2025). Furthermore, HO-1, a heat shock protein, is significantly upregulated in response to oxidative stress or other pathological conditions (Li et al., 2023). The Keap1/Nrf2/HO-1 pathway has been established as a critical antioxidant stress signaling pathway (Guzelcicek et al., 2021; Li et al., 2025). Research indicates (Han et al., 2026) that puerarin mitigates reactive oxygen species (ROS) by activating AMPK, which relieves Keap1’s inhibition of Nrf2 and upregulates the expression of antioxidant enzymes. Concurrently, puerarin inhibits the NF-κB pathway, thereby reducing inflammation and disrupting the detrimental cycle of oxidative stress and inflammation, ultimately alleviating liver damage associated with NAFLD. Additionally, another study demonstrates (Dong et al., 2024) that puerarin, the principal active component of Pueraria lobata extract (APL), exerts antioxidant effects by directly binding to and activating the Keap1/Nrf2/HO-1 pathway. Notably, this effect can be reversed by the Nrf2 inhibitor ML385, suggesting that this pathway is the primary mechanism through which puerarin reduces oxidative stress, improves lipid metabolism disorders, and alleviates NAFLD.

**FIGURE 15:**
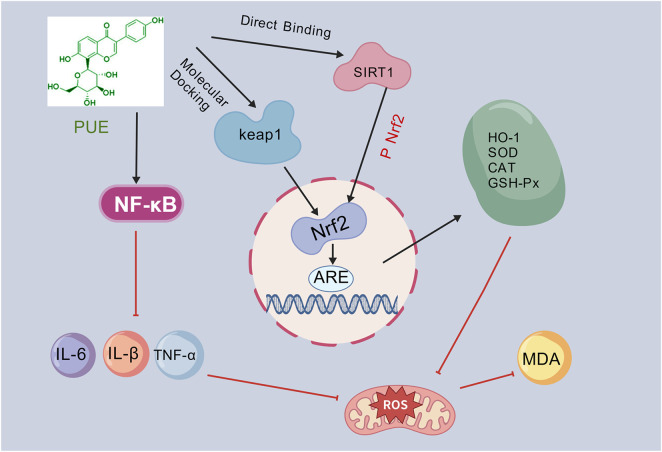
Antioxidant mechanism of puerarin.

SIRT1 is an NAD^+^-dependent deacetylase that plays a crucial role in regulating energy metabolism, mitochondrial biosynthesis, inflammatory responses, and oxidative stress (Tang, 2016; Yang et al., 2022). Previous studies have established that SIRT1 can modulate Nrf2 activity through deacetylation, thereby mitigating oxidative stress (Shen et al., 2025). Mechanistic investigations have demonstrated (Yang et al., 2023) that puerarin directly interacts with the CAP domain of the SIRT1 protein, significantly enhancing its deacetylase activity by forming hydrophobic interactions or hydrogen bonds with amino acid residues such as PRO271 and GLN345. Activated SIRT1 facilitates the translocation of Nrf2 to the nucleus by promoting its deacetylation and p-Nrf2. Once in the nucleus, Nrf2 can upregulate the expression of antioxidant enzymes, effectively eliminating ROS and reducing the generation of MDA. Through this pathway, puerarin can synergistically enhance the antioxidant defense capacity of liver cells and alleviate oxidative stress damage associated with NAFLD (Figure 15).

### Comparative analysis of puerarin and first-line pharmacotherapies

In the current therapeutic landscape, Puerarin demonstrates a unique “multi-target” advantage over single-target synthetic drugs. Unlike Resmetirom—the first FDA-approved THR-β agonist for NASH that primarily focuses on hepatic lipid reduction—Puerarin exerts systemic benefits by simultaneously modulating the gut microbiota-metabolic axis, activating the Keap1/Nrf2/HO-1 antioxidant pathway, and restoring intestinal barrier integrity.

Compared to berberine, another potent natural monomer, our data indicate that Puerarin offers superior efficacy in optimizing lipoprotein profiles, particularly in elevating HDL-C levels (SMD: 2.12; 95% CI: 0.67, 3.57).

Moreover, while statins effectively reduce lipids but carry the risk of elevating hepatic enzymes, Puerarin concurrently mitigates hepatocellular injury during lipid reduction, as evidenced by the synchronous decline in serum ALT and AST (SMD: −3.48 and −2.87, respectively). This comprehensive efficacy validates the “holistic perspective” of TCM and highlights Puerarin’s clinical potential as a multi-dimensional intervention strategy for NAFLD.

### Integration of puerarin pharmacology with TCM clinical practice

NAFLD is typically categorized under “Damp-Heat” or “Phlegm-Stasis” syndromes, with the core pathogenesis centered on the “failure of Yang-Qi to ascend” and the accumulation of metabolic “turbid Qi”. As the primary active isoflavone of Kudzu root, Puerarin has been traditionally utilized to “elevate Yang-Qi” and alleviate metabolic symptoms. Our meta-analysis provides modern pharmacological validation for these classical concepts. Specifically, the “elevation of Yang-Qi” scientifically correlates with the activation of AMPK/SREBP-1c and PPAR-α pathways observed in our results, which “reboot” hepatic energy metabolism and fatty acid β-oxidation.

Furthermore, the clinical application of Kudzu root in TCM formulae, such as Gegen Qinlian Decoction, highlights its roles in “clearing heat” and regulating “intestinal function”. This aligns with our findings regarding the gut microbiota-metabolism axis: Puerarin effectively “clears” endogenous inflammatory “heat” triggered by high-fat and high-sugar diets by repairing the intestinal barrier and reducing LPS translocation.

### Clinical relevance of experimental models to the Chinese population

To contextualize our findings for the Chinese population, we stratified preclinical models into HFD, HFFD, and HFHSD subgroups. The results confirmed the robust efficacy of Puerarin across diverse diet-induced NAFLD models (Supplementary Figures 12, 16, 20, 24, 28, 32). Notably, in HFFD and HFHSD models—which simulate the modern Chinese transition toward high-fructose and high-carbohydrate intake—Puerarin maintained significant improvements in hepatic transaminases and lipid accumulation (p < 0.05). By synthesizing TCM theory with multi-target systemic pharmacology, this study establishes a coherent evidence-based framework that bridges regional clinical traditions with global therapeutic standards.

### Limitations

This study presents evidence supporting the clinical application of puerarin in the treatment of NAFLD through preclinical evaluation, while also recognizing its limitations.

### Limitations of methodological

Based on the GRADE assessment, the certainty of preclinical evidence for puerarin in treating NAFLD remains low. This is primarily due to the lack of blinded designs and significant statistical heterogeneity, necessitating caution when translating these mechanistic findings into clinical practice.

This limited evidence quality stems from methodological opacity in the included studies. Specifically, the absence of detailed reporting on randomization methods, allocation concealment, and outcome-assessor blinding introduces potential performance and detection biases, thereby undermining the overall reliability of the synthesized evidence.

To enhance the rigor of future translational research, we strongly recommend that future preclinical studies strictly adhere to the ARRIVE (Animal Research: Reporting of *In Vivo* Experiments) guidelines. Compliance with these standards—particularly regarding precise randomization, rigorous blinding, and *a priori* sample size calculation—is essential to ensure transparency. Standardizing experimental design and reporting will fundamentally improve reproducibility and provide a more robust scientific foundation for the clinical application of puerarin.

### Limitations of animal sex representation

Another critical limitation of the current evidence base is the overwhelming reliance on male animals, which neglects Sex as a Biological Variable (SABV). NAFLD exhibits significant sexual dimorphism in its pathophysiology; for instance, endogenous estrogen provides potent protection for hepatic lipid metabolism and systemic insulin sensitivity, often leading to disparities in disease prevalence and progression between sexes.

Furthermore, given that Puerarin is an isoflavone with established phytoestrogenic properties, its therapeutic efficacy and underlying mechanisms may vary significantly depending on the hormonal environment. Consequently, the near-exclusive use of male models—with only one study utilizing females—limits the generalizability of our meta-analysis findings. Future preclinical investigations must include both male and female subjects to elucidate potential sex-specific therapeutic responses and ensure a comprehensive evaluation of Puerarin’s clinical translational potential.

### Limitations of pharmacokinetics and interspecies disparities

The pharmacokinetic (PK) and interspecies differences warrant critical consideration. Although the prevalent use of Sprague-Dawley rats and C57BL/6 mice in the included studies provides high-fidelity data relevant to the Chinese drug development context, significant disparities exist between rodents and humans regarding metabolic rates, bioavailability, and PK profiles. Consequently, the metabolic clearance and target sensitivity of Puerarin in animals may not fully replicate the complex human environment. Furthermore, the current emphasis on short-term efficacy rather than long-term, low-dose exposure creates uncertainty in clinical dose extrapolation. Future research should integrate physiologically based pharmacokinetic (PBPK) modeling, human liver organoids, or humanized mouse models to more accurately simulate the metabolic characteristics and therapeutic responses specific to Chinese NAFLD patients.

### Potential confounding factors

Despite preliminary insights from subgroup analyses, several potential confounders may still affect the reliability of these findings. First, animal age across the included models was highly concentrated between 6 and 8 weeks. While this standardization ensures a uniform metabolic state in early adulthood and enhances the comparability of effect sizes by minimizing developmental variance, it limits the generalizability of the conclusions to other life stages, such as geriatric or pediatric populations. Second, the source, purity, and preparation of Puerarin were inconsistently reported, and the administration route was exclusively restricted to oral gavage. These factors may obscure the impact of differing bioavailability on therapeutic outcomes. Consequently, future preclinical studies should maintain experimental standardization while enhancing the transparent reporting of key confounders—such as genetic sub-strains, environmental enrichment, and dosing precision—to strengthen the evidence base for clinical translation.

### Challenges and pathways for clinical translation

#### Dose extrapolation and the therapeutic window

We employed the Body Surface Area (BSA) normalization method to estimate the Human Equivalent Dose (HED). Based on the effective animal dosage range identified in this meta-analysis (100–400 mg/kg), the estimated HED for a 60 kg adult is approximately 8–32 mg/kg. This quantification provides a rational pharmacological starting point for Phase I clinical trials and the establishment of a preliminary therapeutic window.

#### Overcoming bioavailability and pharmacokinetic barriers

We have incorporated a critical discussion regarding the low oral bioavailability of puerarin. Despite its significant efficacy in rodent models, puerarin faces substantial translational challenges in humans due to poor intestinal absorption and rapid first-pass metabolism. Future research must prioritize structural modifications or innovative delivery systems, such as nano-formulations, to circumvent these pharmacological hurdles. Enhancing bioavailability is a prerequisite for translating “potent animal performance” into “robust human efficacy”.

#### Drug-drug interactions (DDIs) in complex metabolic contexts

Given that patients with NAFLD often present with multiple metabolic comorbidities, we explored potential DDIs between puerarin and standard-of-care pharmacotherapies, such as statins or metformin. Our findings highlight puerarin’s therapeutic advantage: it concurrently reduces lipids and repairs hepatocellular injury (evidenced by the synchronous decline in ALT and AST levels). This suggests favorable safety compatibility and potential synergistic hepatoprotective effects in combination regimens.

### Evidence-based recommendations for clinical trial design

While large-scale Randomized Controlled Trials (RCTs) using puerarin monotherapy are currently lacking, Puerarin-containing TCM formulae, such as the Gegen Qinlian Decoction, have demonstrated definitive clinical efficacy in improving NAFLD and insulin resistance. This historical clinical consistency provides a solid “clinical starting point” for the translation of puerarin monomer into modern hepatology. The robust performance of puerarin in optimizing lipid metabolism and reducing transaminases, as validated by this meta-analysis, aligns closely with observed clinical benefits in metabolic disorders. Future clinical trials should prioritize patients with pathologically or radiologically confirmed NAFLD. Primary endpoints should focus on sustained improvements in serum transaminases or reductions in hepatic steatosis (e.g., via MRI-PDFF). Preclinical data indicate that puerarin typically requires 16 days to 8 weeks to significantly alleviate steatosis, a time-dependent efficacy profile that provides a critical basis for defining appropriate clinical observation periods and follow-up intervals in human trials. Integrating these preclinical insights into clinical protocols will facilitate a more efficient evaluation of puerarin’s therapeutic potential.

### Future perspectives

While this meta-analysis underscores the significant hepatoprotective potential of puerarin, bridging the gap from bench to bedside requires addressing several critical research lacunae. First, as current evidence relies predominantly on short-term interventions (4–8 weeks), future studies should prioritize longitudinal models to evaluate its long-term efficacy and safety, particularly regarding its sustained impact on liver fibrosis and cirrhosis. Second, there is an urgent need for research on combination therapies—pairing puerarin with first-line drugs (e.g., metformin, statins) or weight-loss interventions—to explore synergistic effects within a “multiple-hit” management framework. Furthermore, future studies must differentiate between clinico-pathological subtypes of NAFLD to delineate stage-specific target specificity across simple steatosis, NASH, and fibrosis. Mechanistically, research should evolve beyond classical pathways to integrate cutting-edge processes such as mitochondrial dysfunction, autophagy, and pyroptosis, while leveraging standardized purification and nano-delivery systems to enhance bioavailability. Notably, as the field transitions toward the MASLD framework, puerarin’s dual benefits in alleviating hepatic steatosis and systemic metabolic dysregulation align perfectly with this new paradigm, highlighting its substantial translational value in targeting metabolism-driven liver disorders.

## Data Availability

The original contributions presented in the study are included in the article/Supplementary Material, further inquiries can be directed to the corresponding author.
