# Supplementary material for: Preclinical evaluation of puerarin for the treatment of non-alcoholic fatty liver disease: a systematic review and meta-analysis
Source: Front Pharmacol. 2026 Apr 13;17:1795374. doi: 10.3389/fphar.2026.1795374 (PMC13111457; doi:10.3389/fphar.2026.1795374)
Supplement: Supplementary file 1 [file Presentation1.zip › Revised Supplementary_Material.docx]

Supplementary Material

# search strategy

# Pubmed :

# ((Non-alcoholic Fatty Liver Disease [MeSH Terms]) OR (Non alcoholic Fatty Liver Disease [Title/Abstract]) OR (NAFLD [Title/Abstract]) OR (Nonalcoholic Fatty Liver Disease [Title/ Abstract]) OR (Fatty Liver, Nonalcoholic [Title/Abstract]) OR(Fatty Livers,Nonalcoholic [Title/Abstract]) OR (Liver, Nonalcoholic Fatty [Title/Abstract]) OR (Livers, Nonalcoholic Fatty [Title/Abstract]) OR (Nonalcoholic Fatty Liver [Title/Abstract]) OR (Nonalcoholic Fatty Livers [Title/Abstract]) OR (Nonalcoholic Steatohepatitis [Title/Abstract])OR (Steatohepatitides, Nonalcoholic [Title/Abstract]) OR (Steatohepatitis, Nonalcoholic [Title/Abstract])) AND ((Puerarin [MeSH Terms]) OR (Puerarin [Title/Abstract]) OR (Kudzu [Title/Abstract]) OR (Kudzu [Title/Abstract]) OR (Kudzu root extract puerarin[Title/Abstract]) OR (Kudzu root extract puerarin [Title/Abstract]))

# web of science :

# (TS=("Non-alcoholic Fatty Liver Disease" OR "Non-alcoholic Steatohepatitis" OR "Fatty Liver, Non-alcoholic") OR TS=NAFLD OR TS=NASH OR TS="Non-alcoholic fatty liver disease" OR TS="Nonalcoholic fatty liver disease" OR TS="Non-alcoholic steatohepatitis" OR TS="Nonalcoholic steatohepatitis") AND (TS=Puerarin OR TS=Kudzu OR TS="Puerarin 8-beta-D-glucoside" OR TS="Daidzein 8-C-glucoside" OR TS="Kudzu root extract puerarin")

# Embase :

# #1 Non alcoholic Fatty Liver Disease

# #2 'Non alcoholic Fatty Liver Disease':ab,kw,

# #3 'Non alcoholic Fatty Liver Disease':ab,kw,ti OR 'NAFLD':ab,kw,ti OR 'Nonalcoholic Fatty Liver Disease':ab,kw,ti OR 'Fatty Liver, Nonalcoholic':ab,kw,ti OR 'Fatty Livers, Nonalcoholic':ab,kw,ti OR 'Liver, Nonalcoholic Fatty':ab,kw,ti OR 'Livers, Nonalcoholic Fatty':ab,kw,ti OR 'Nonalcoholic Fatty Liver':ab,kw,ti OR 'Nonalcoholic Fatty Livers':ab,kw,ti OR 'Nonalcoholic Steatohepatitis':ab,kw,ti OR 'steatohepatitis, nonalcoholic':ab,kw,ti OR 'Steatohepatitis, Nonalcoholic':ab,kw,ti

# #4 Puerarin

# #5 'Puerarin':ab,kw,

# #6 'Puerarin':ab,kw,ti OR 'Kudzu':ab,kw,ti OR 'Kudzu root extract puerarin':ab,kw,ti

# #7 # 1OR #3

# #8 #4 OR #6

# #9 #7 AND #8

# Cochrane Library :

# #1 MeSH descriptor: [Non-alcoholic Fatty Liver Disease] explode all trees

# #2 (Fatty Liver, Nonalcoholic OR Non alcoholic Fatty Liver Disease OR Nonalcoholic Fatty Liver OR Livers, Nonalcoholic Fatty OR Nonalcoholic Fatty Liver Disease OR Liver, Nonalcoholic Fatty OR NAFLD OR Fatty Livers, Nonalcoholic OR Nonalcoholic Fatty Livers OR Steatohepatitis, Nonalcoholic OR Steatohepatitides, Nonalcoholic OR Nonalcoholic Steatohepatitides OR Nonalcoholic Steatohepatitis):ti,ab,kw (Word variations have been searched)

# #3 #1 OR #2

# #4 MeSH descriptor: [Pueraria] explode all trees

# #5 (tuberosa, Pueraria OR Pueraria tuberosa OR Pueraria tuberosas OR Pueraria lobatas OR Pueraria lobata OR lobatas, Pueraria OR Puerarias OR Puerariae OR Pueraria montanas OR Pachyrhizus thunbergianus OR Pueraria montana OR montana, Pueraria OR Kudzus OR Kudzu):ti,ab,kw (Word variations have been searched)

# #6 #4 OR #5

# #7 #3 AND #6

# CNKI, Wangfang Data, VPCS,SinoMed :

# Search terms included:“葛根素” (Puerarin);“非酒精性肝病” (Non-alcoholic Fatty Liver Disease)

# Boolean operator AND was used to combine terms. No language restrictions were applied within Chinese databases.

# Supplementary Figures

#
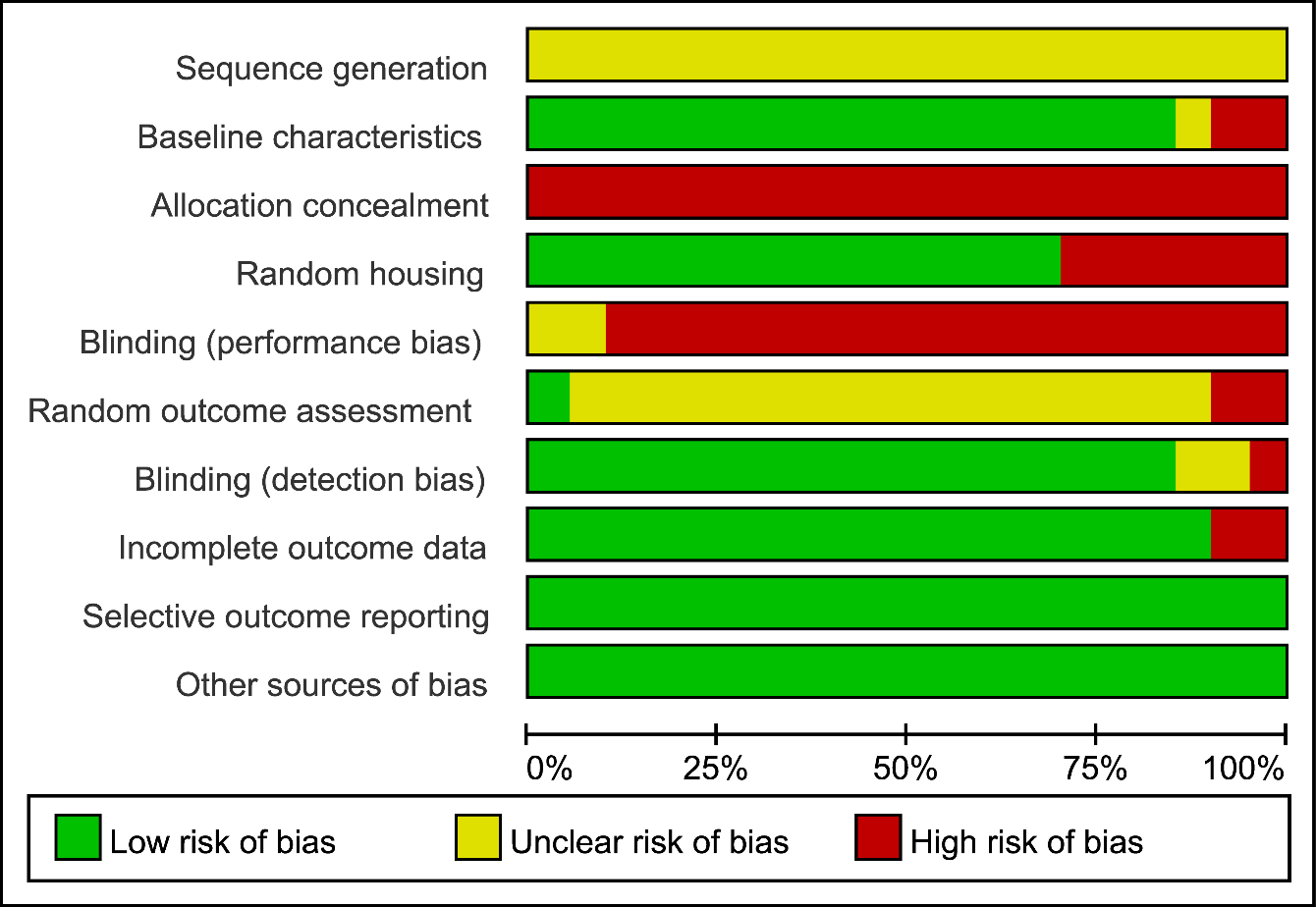


# Supplementary Figure 1. Summary of the Risk of Bias for Included Studies.

#
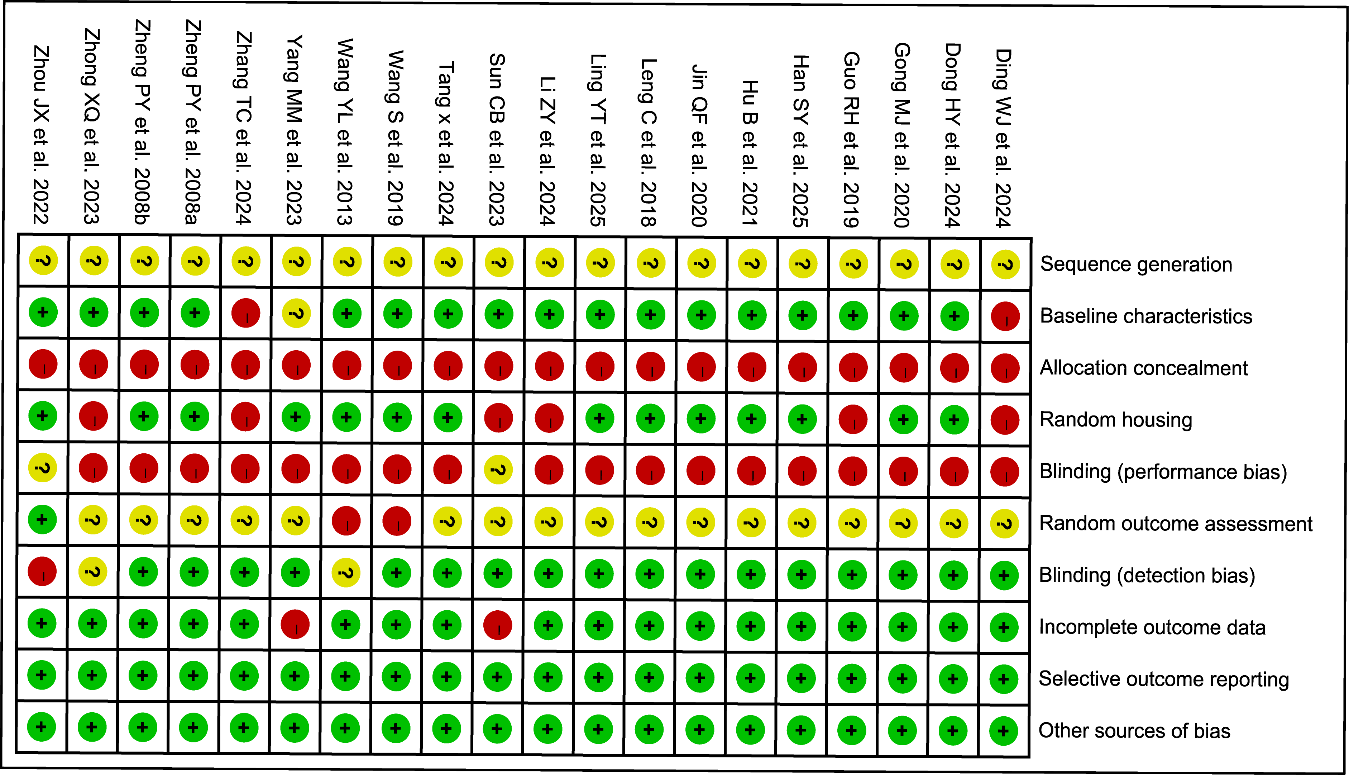


# Supplementary Figure 2. Scatter Plot of the Risk of Bias in Included Studies.

#
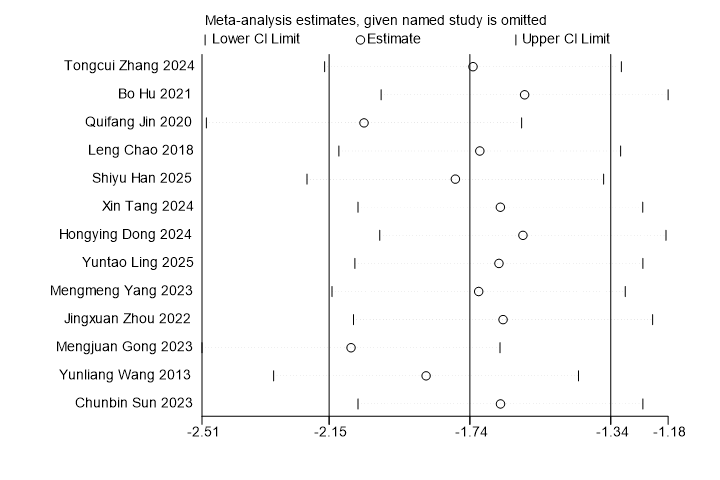


# Supplementary Figure 3. sensitivity analysis of Serum TG

#
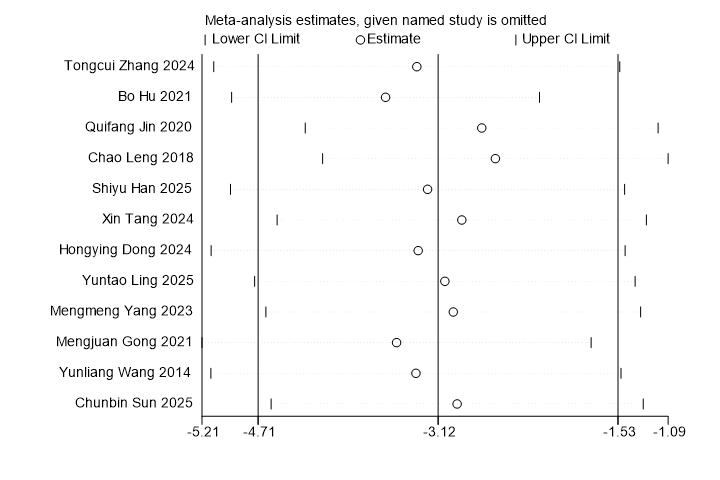


# Supplementary Figure 4. sensitivity analysis of Serum TC

#
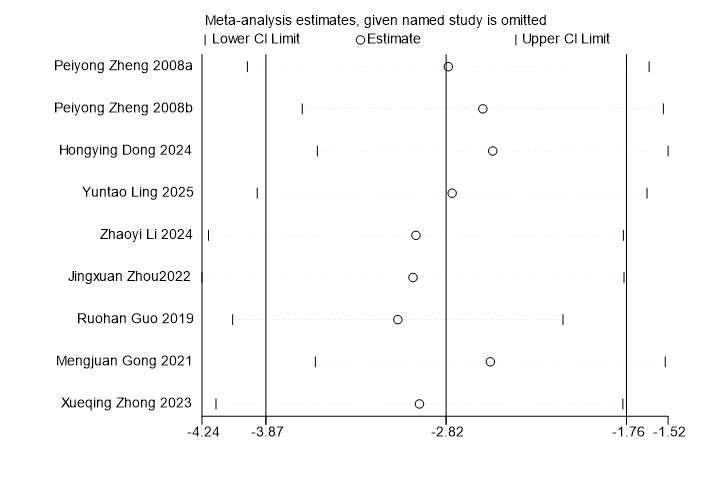


# Supplementary Figure 5. sensitivity analysis of liver TG

#
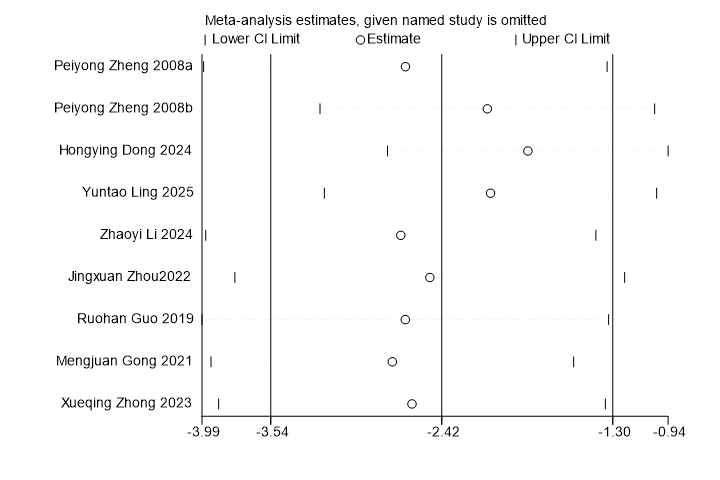


# Supplementary Figure 6. sensitivity analysis of liver TC

#
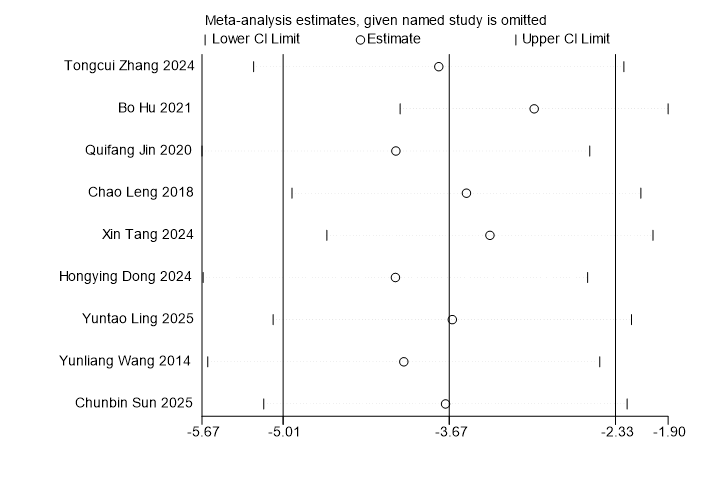


# Supplementary Figure 7. sensitivity analysis of LDL-C

#
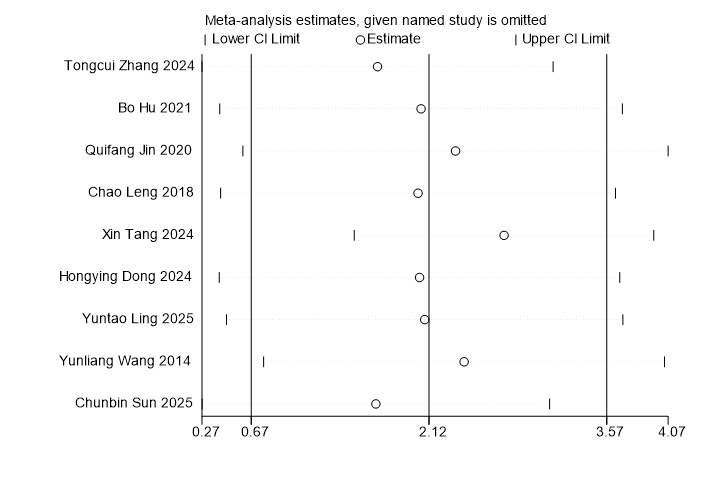


# Supplementary Figure 8. sensitivity analysis of HDL-C

#
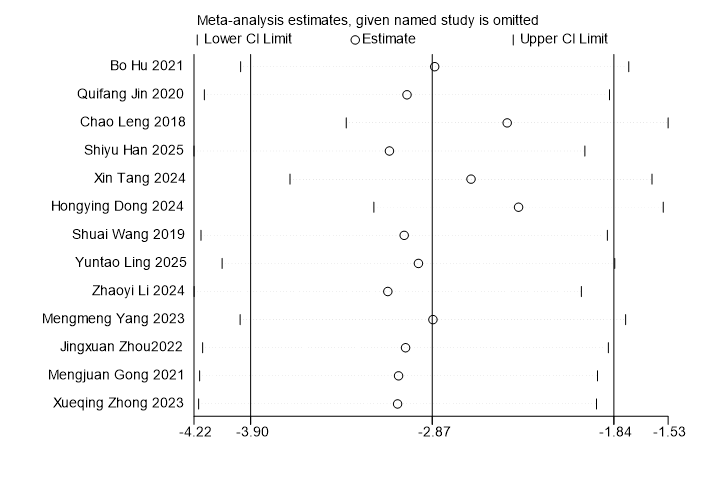


# Supplementary Figure 9. sensitivity analysis of AST

#
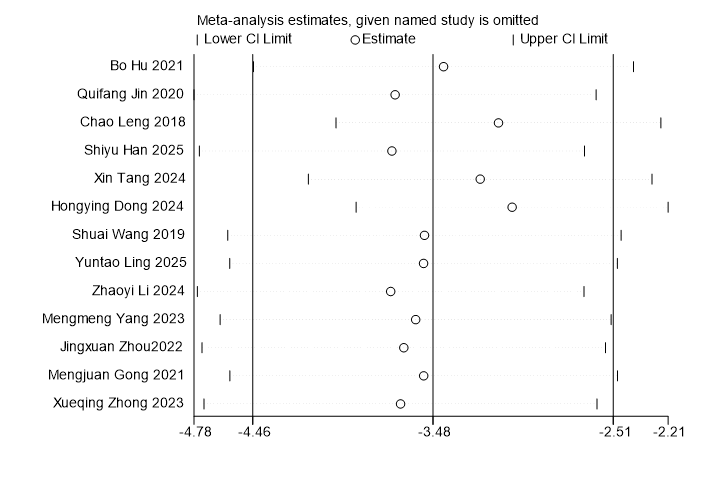


# Supplementary Figure 10. sensitivity analysis of ALT

#
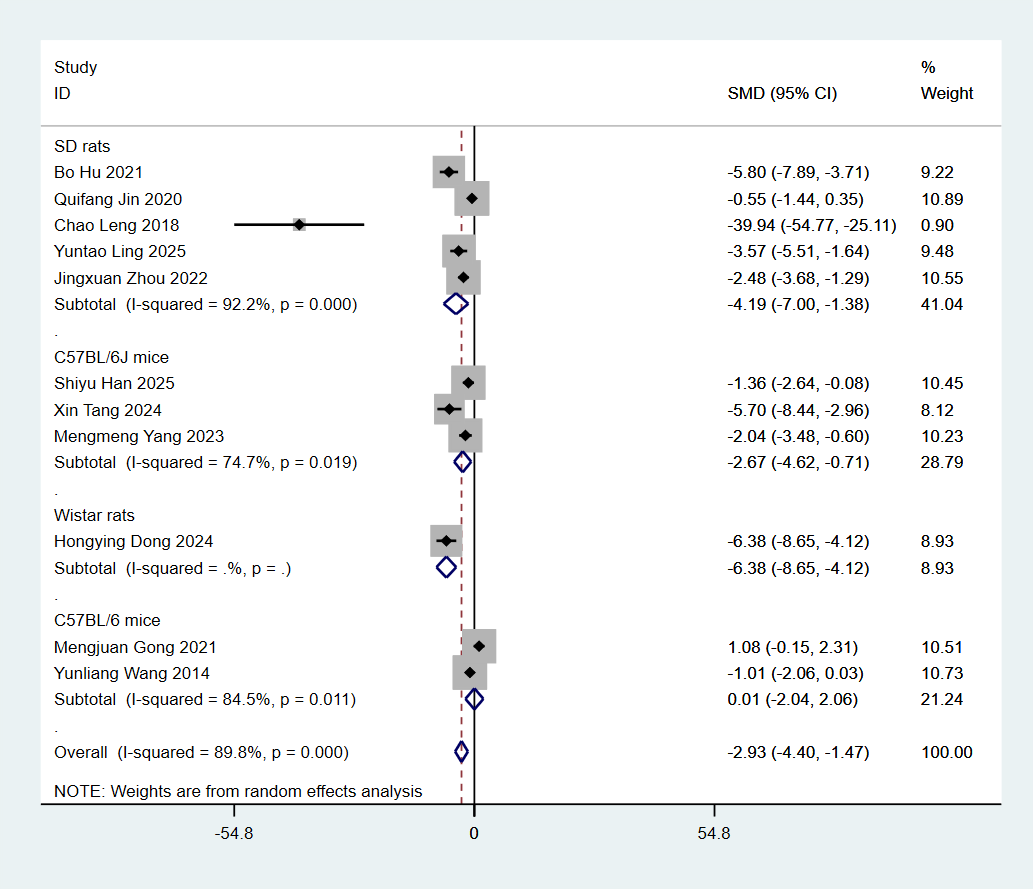


# Supplementary Figure 11. Subgroup analysis of Serum TG based on species of animal

#
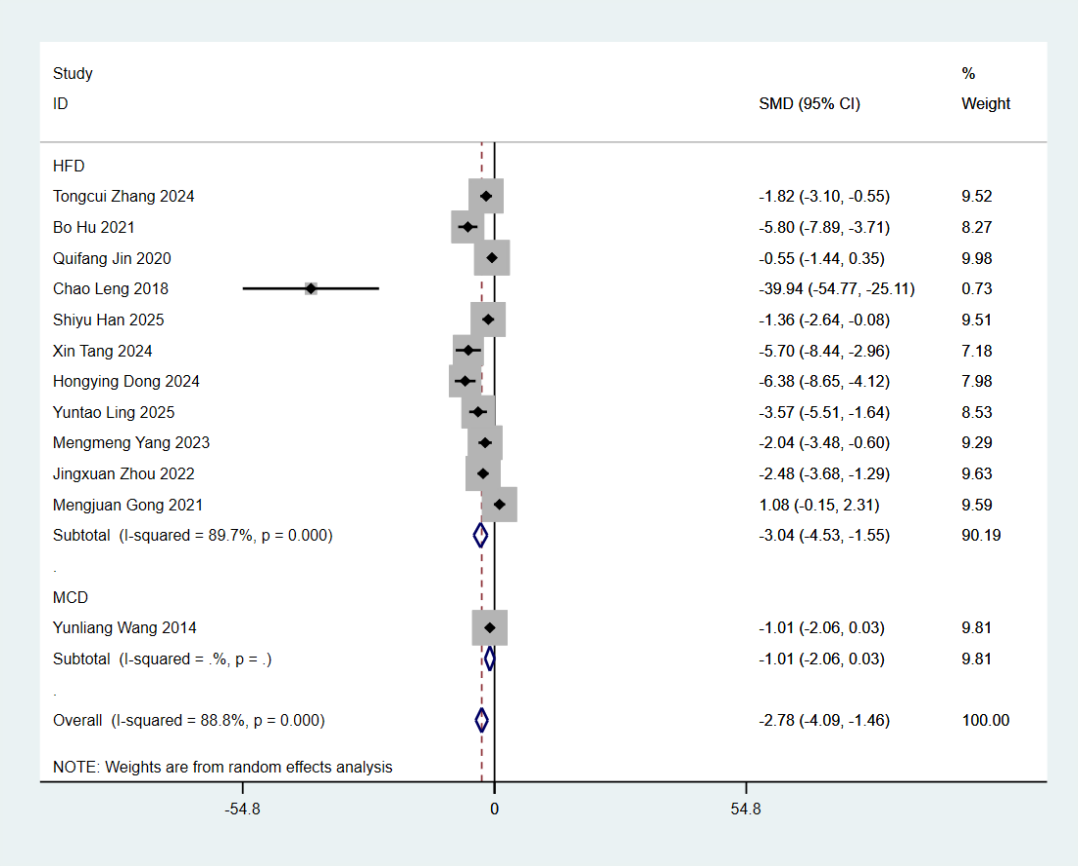


# Supplementary Figure 12. Subgroup analysis of Serum TG based on modeling methodologies

#
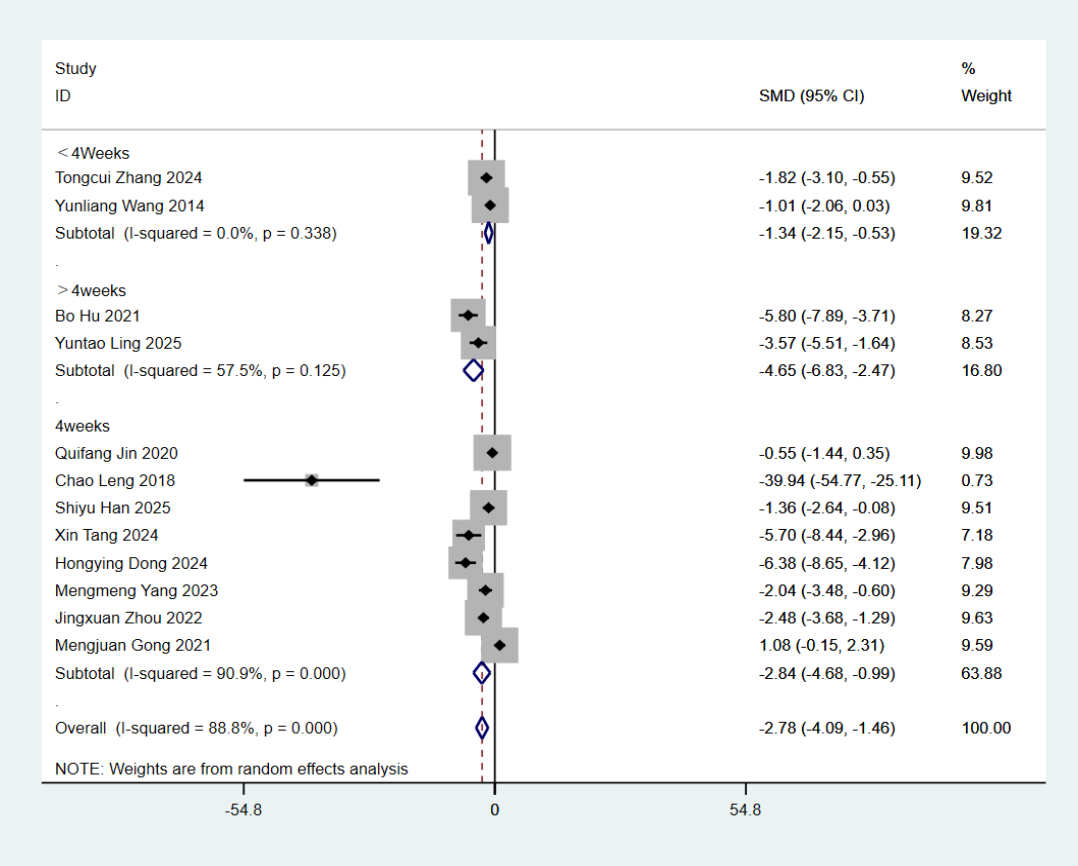


# Supplementary Figure 13. Subgroup analysis of Serum TG based on intervention duration

#
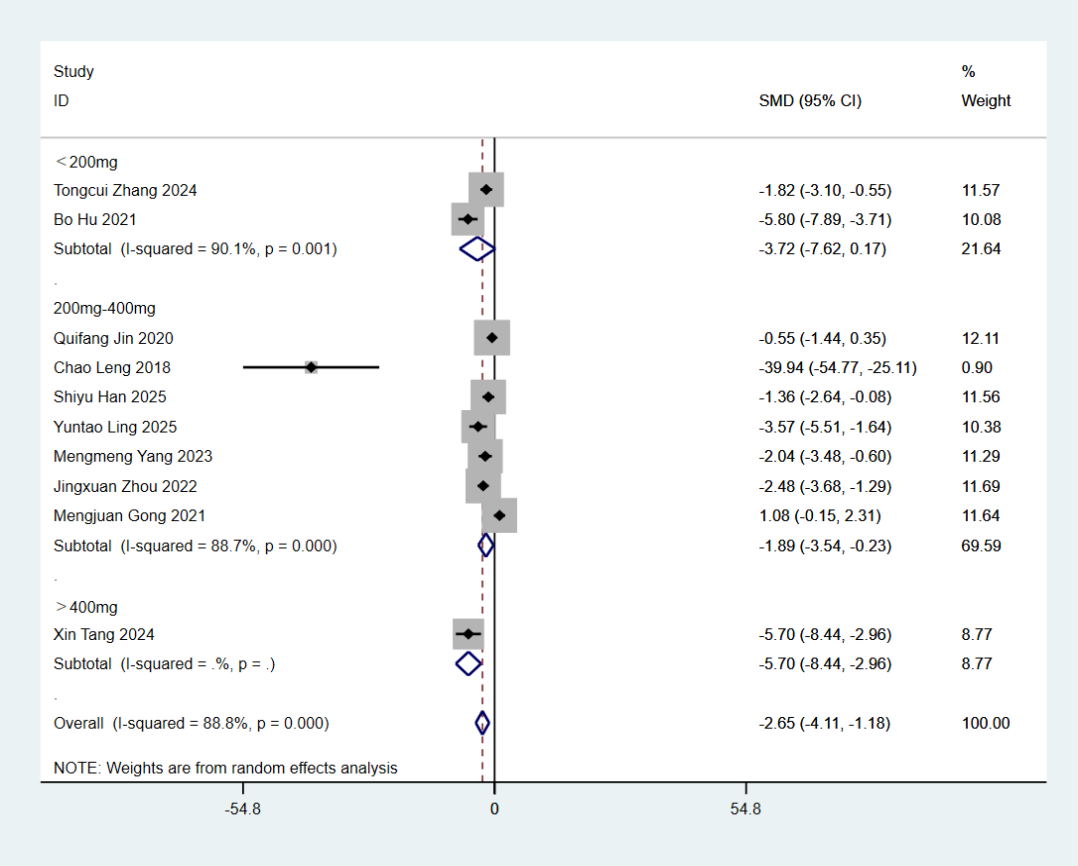


# Supplementary Figure 14. Subgroup analysis of Serum TG based on dosage regimens of puerarin

#
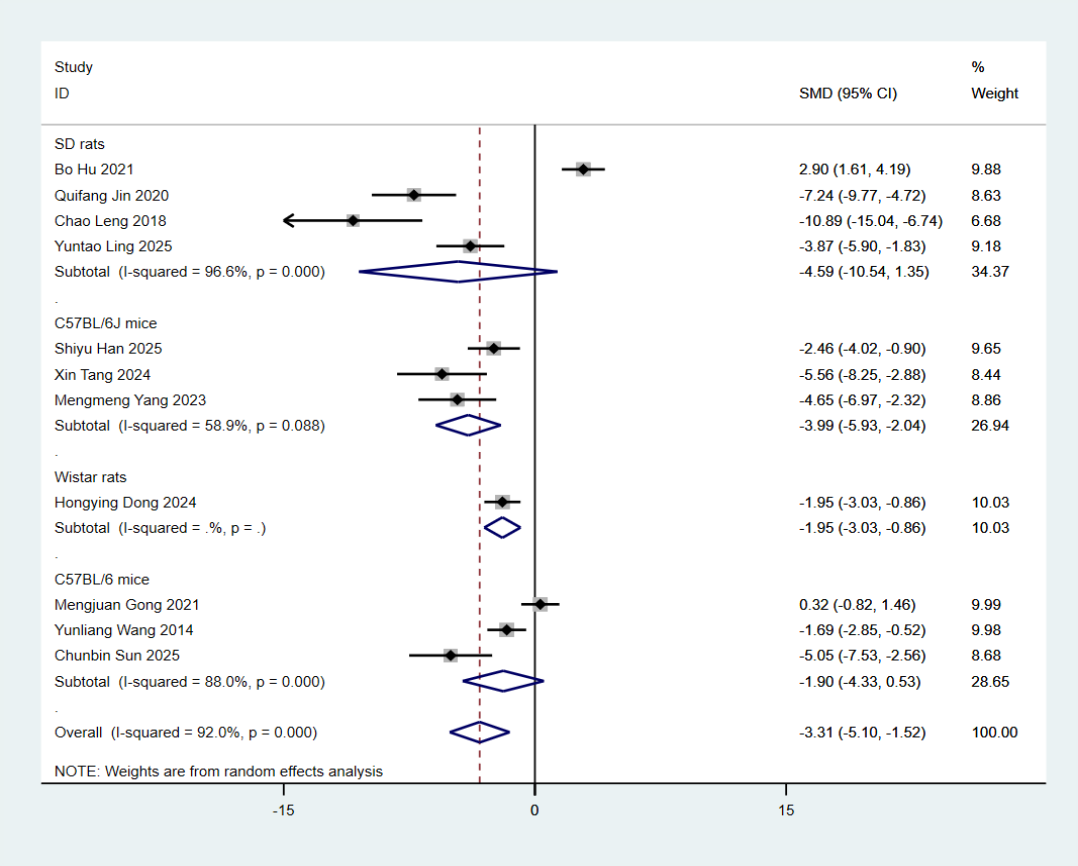


# Supplementary Figure 15. Subgroup analysis of Serum TC based on species of animal

#
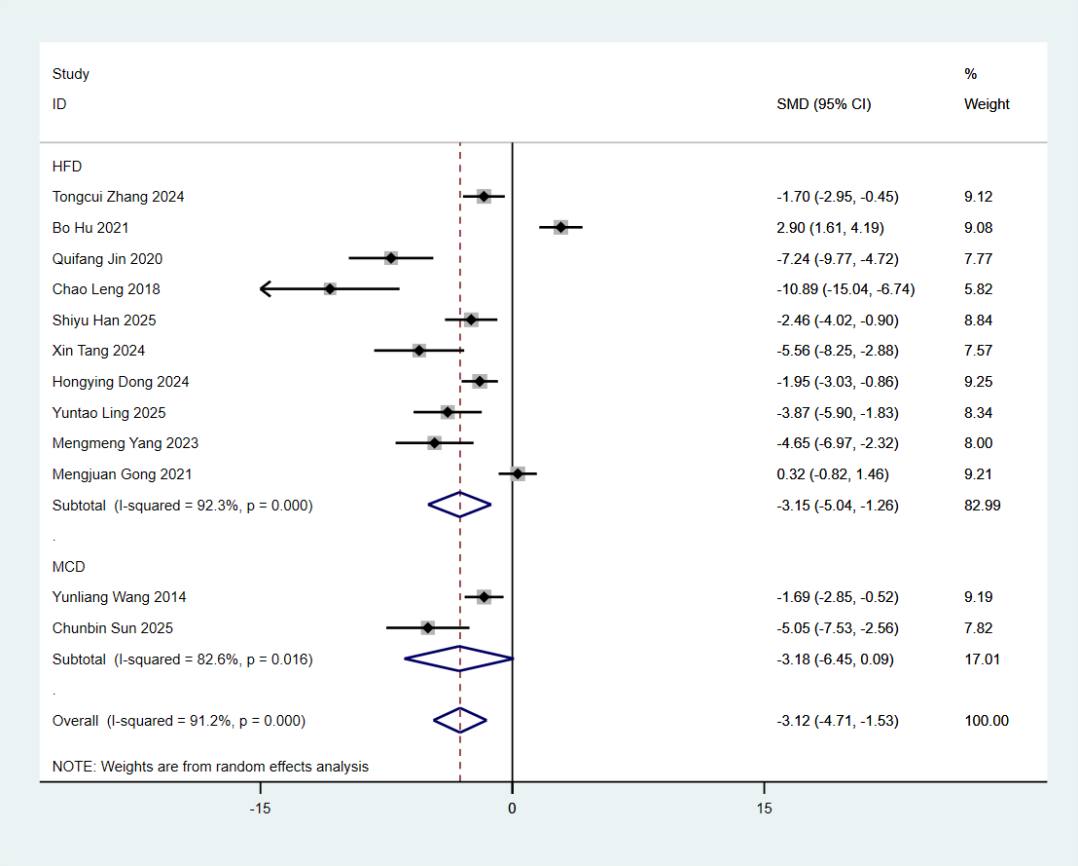


# Supplementary Figure 16. Subgroup analysis of Serum TC based on modeling methodologies

#
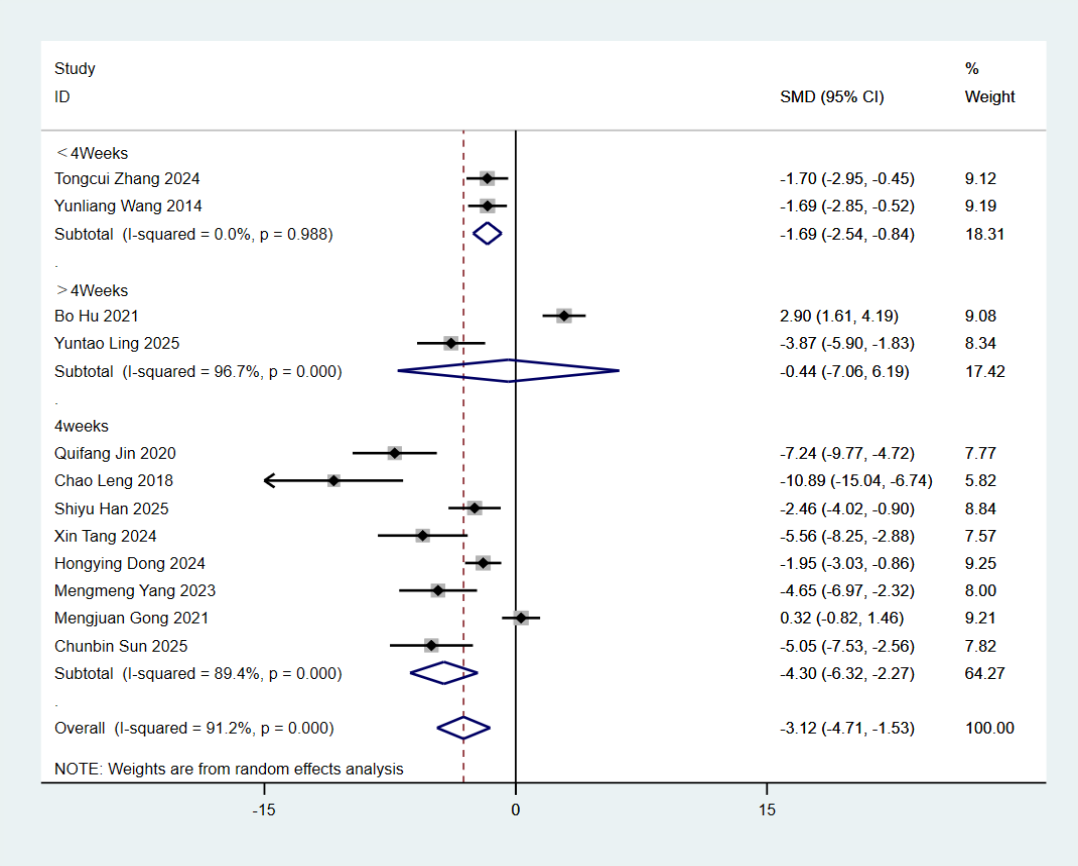


# Supplementary Figure 17. Subgroup analysis of Serum TC based on intervention duration

#
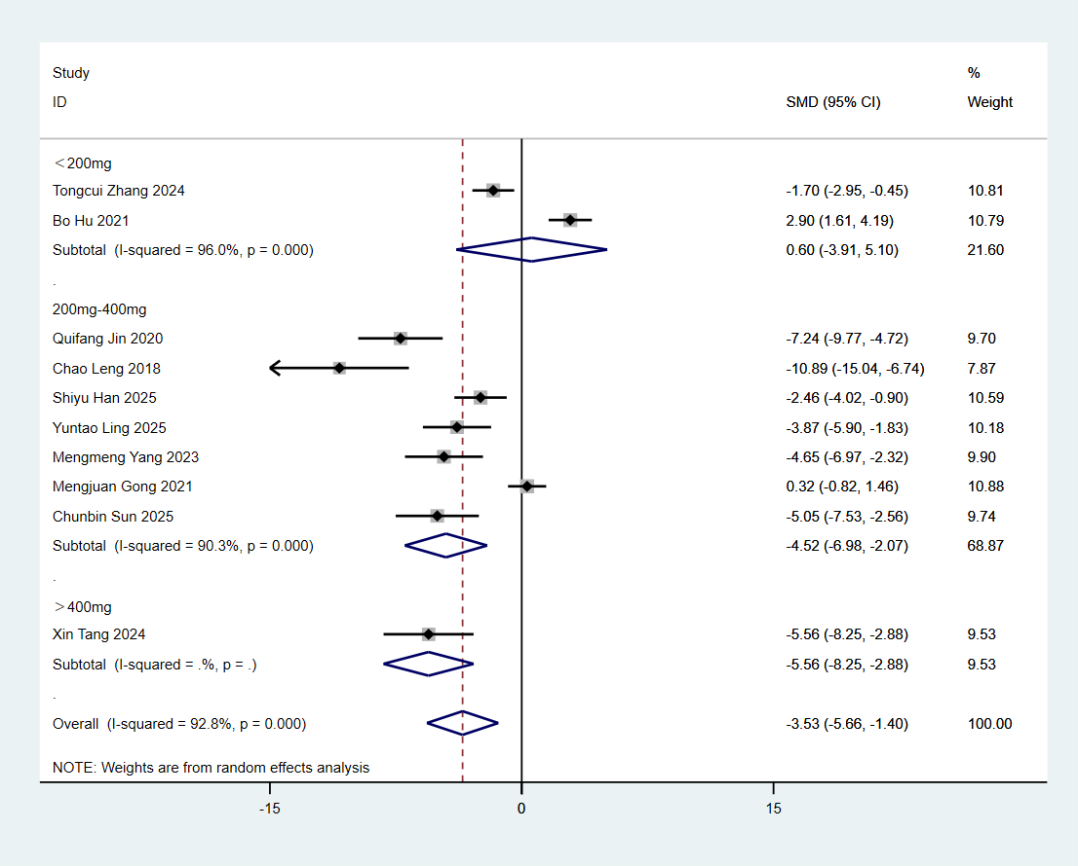


# Supplementary Figure 18. Subgroup analysis of Serumr TG based on dosage regimens of puerarin

#
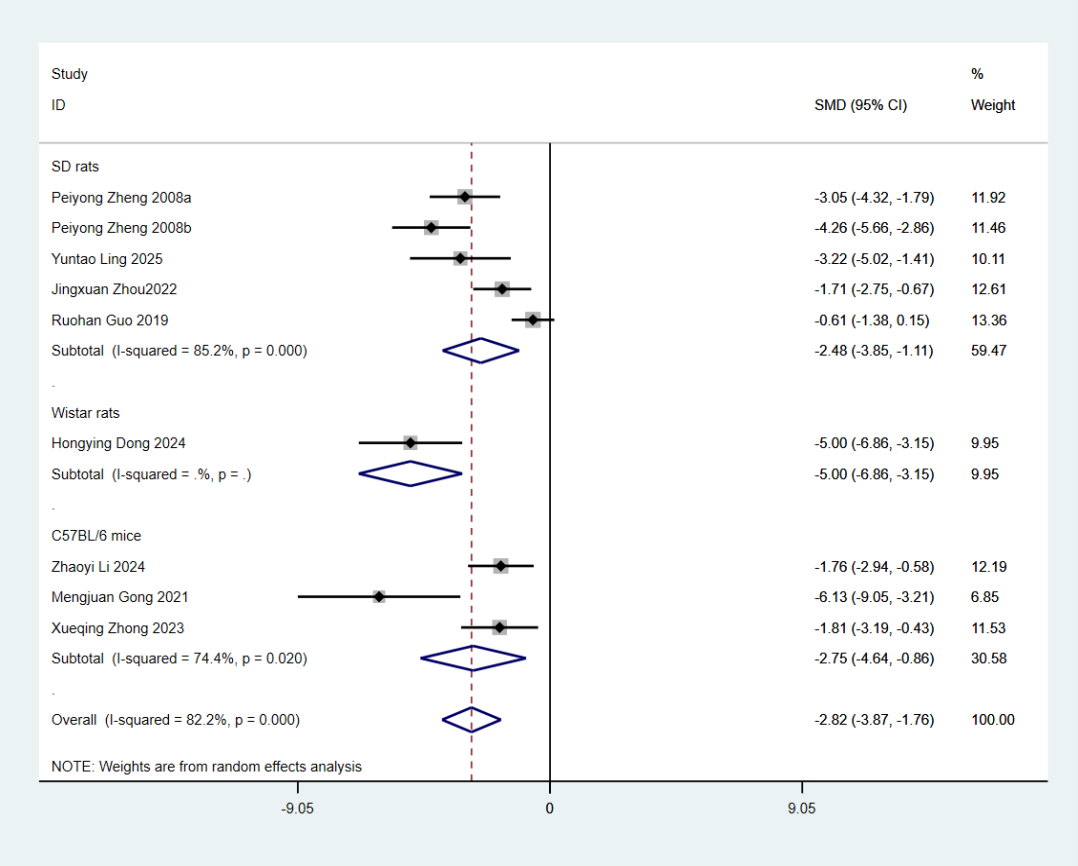


# Supplementary Figure 19. Subgroup analysis of liver TG based on species of animal

#
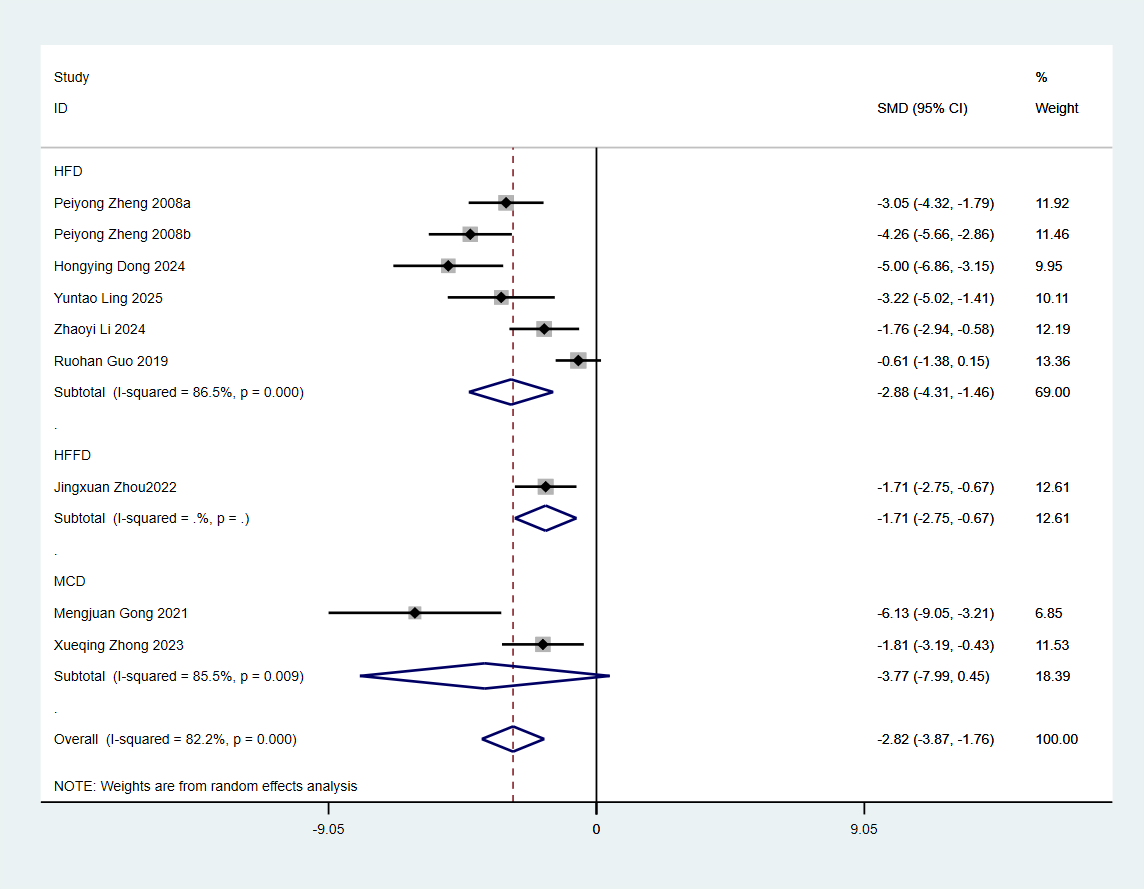


# Supplementary Figure 20. Subgroup analysis of liver TG based on modeling methodologies

#
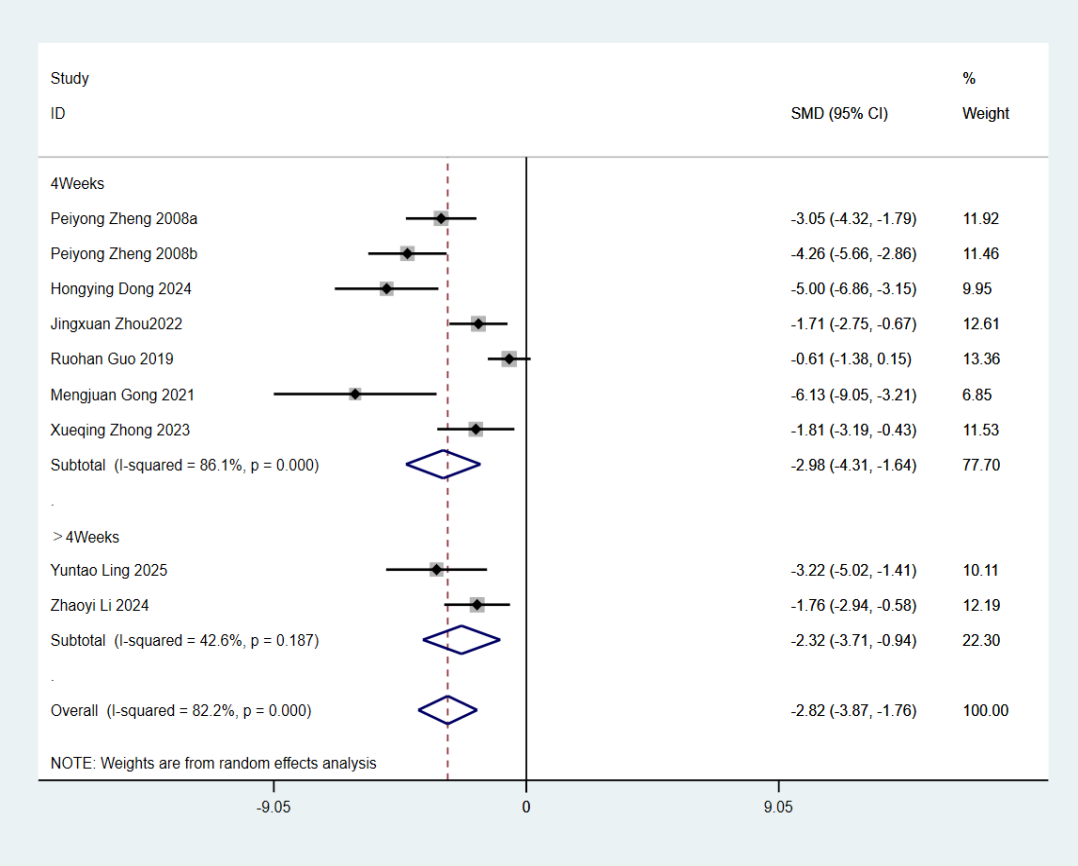


# Supplementary Figure 21. Subgroup analysis of liver TG based on intervention duration

#
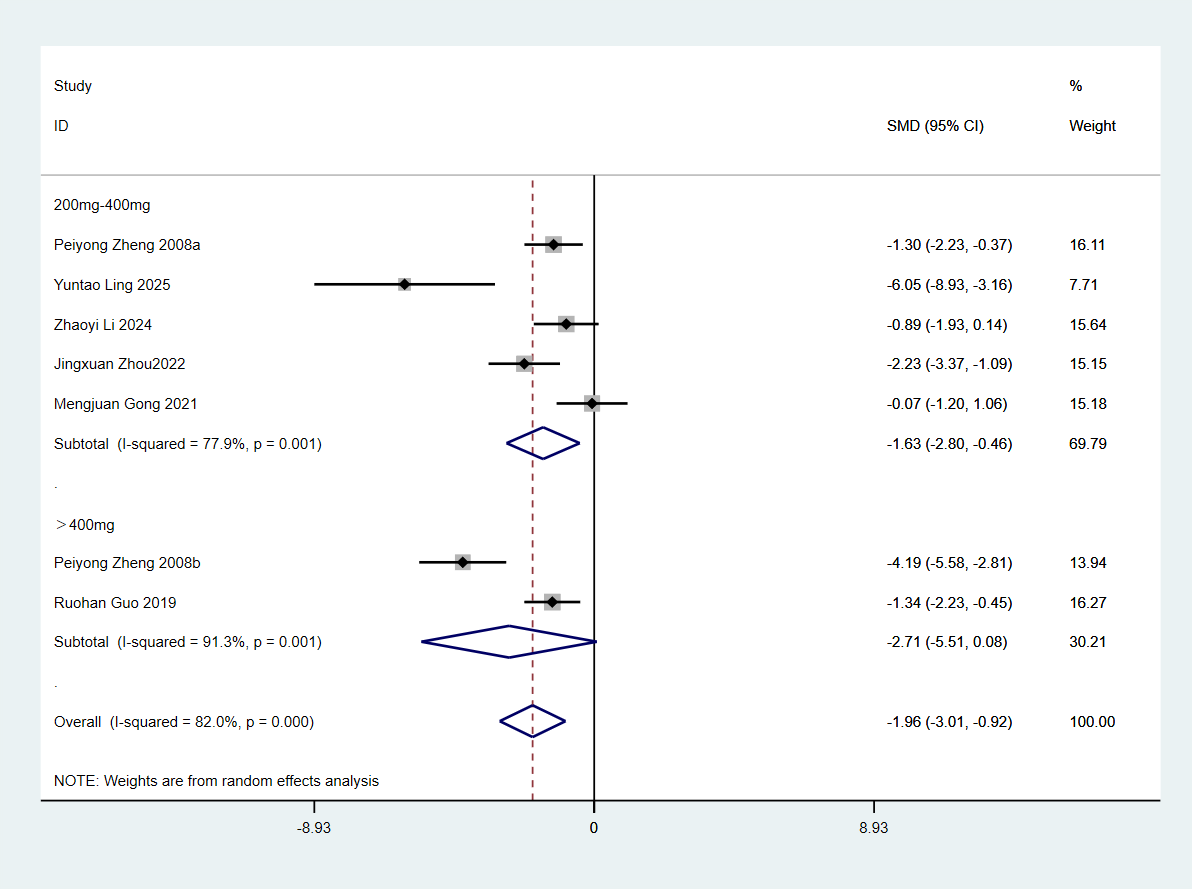


# Supplementary Figure 22. Subgroup analysis of liver TG based on dosage regimens of puerarin

#
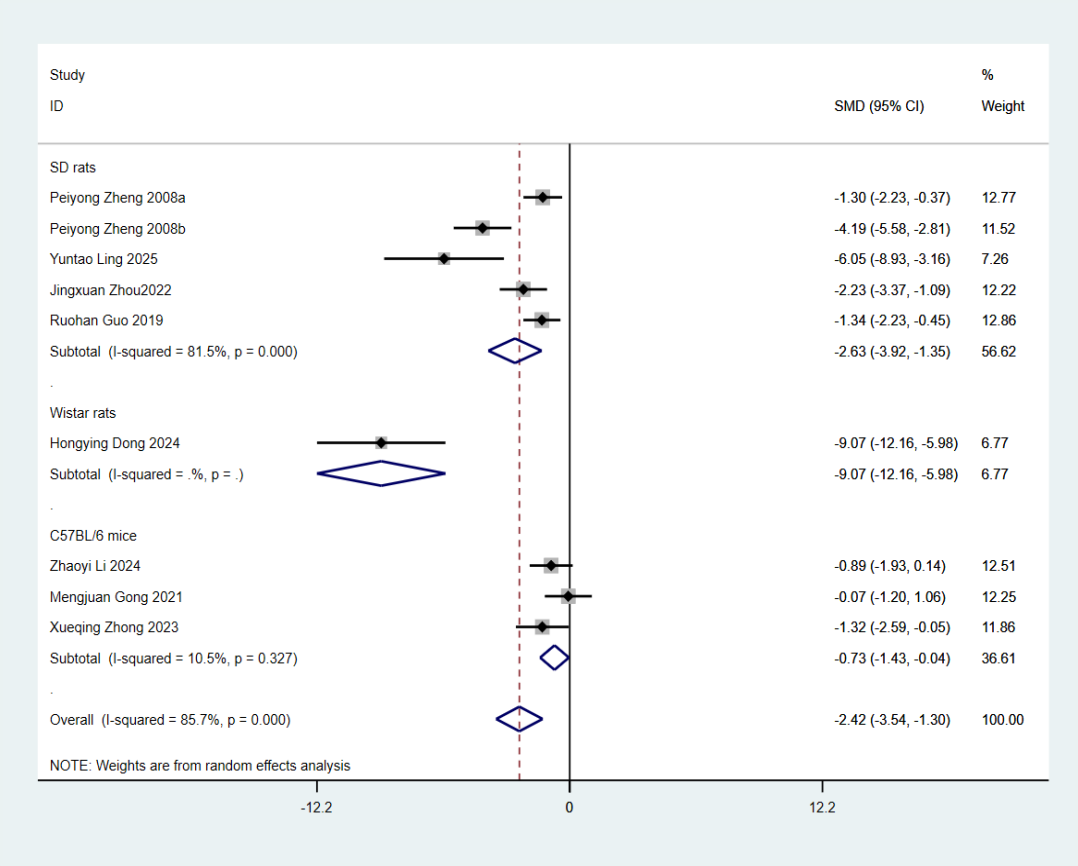


# Supplementary Figure 23. Subgroup analysis of liver TC based on species of animal

#
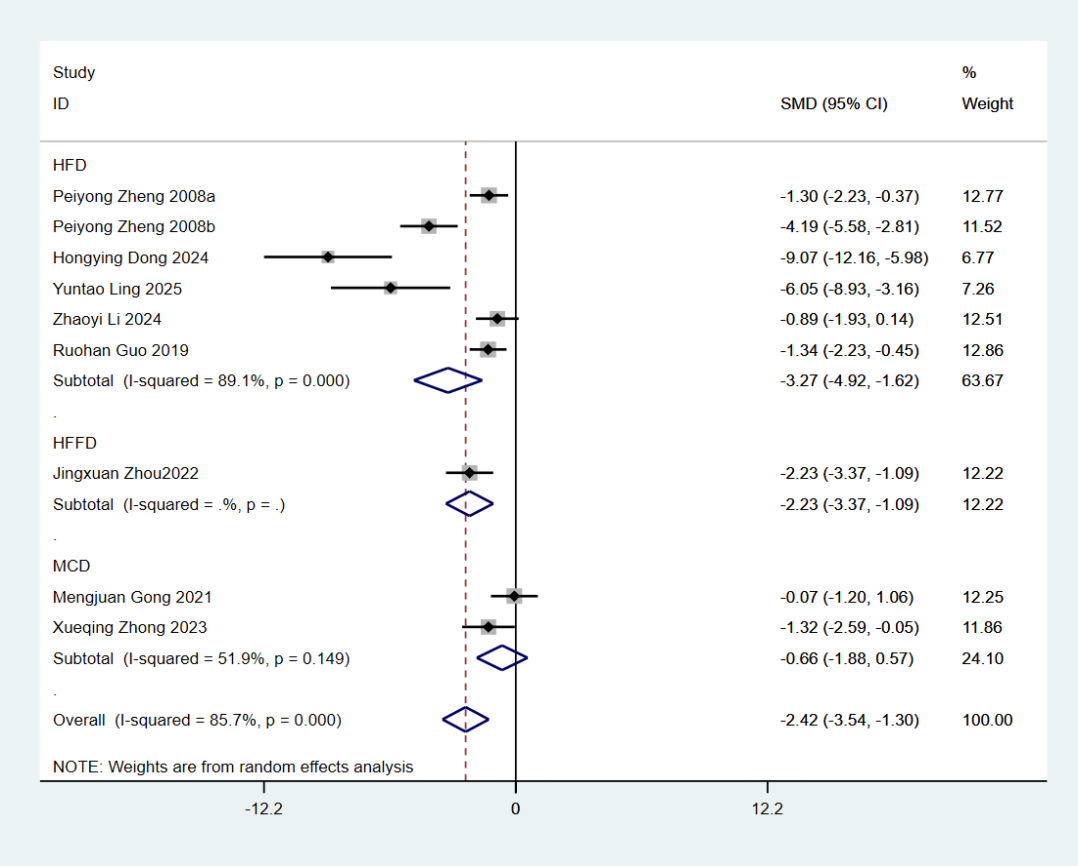


# Supplementary Figure 24. Subgroup analysis of liver TC based on modeling methodologies

#
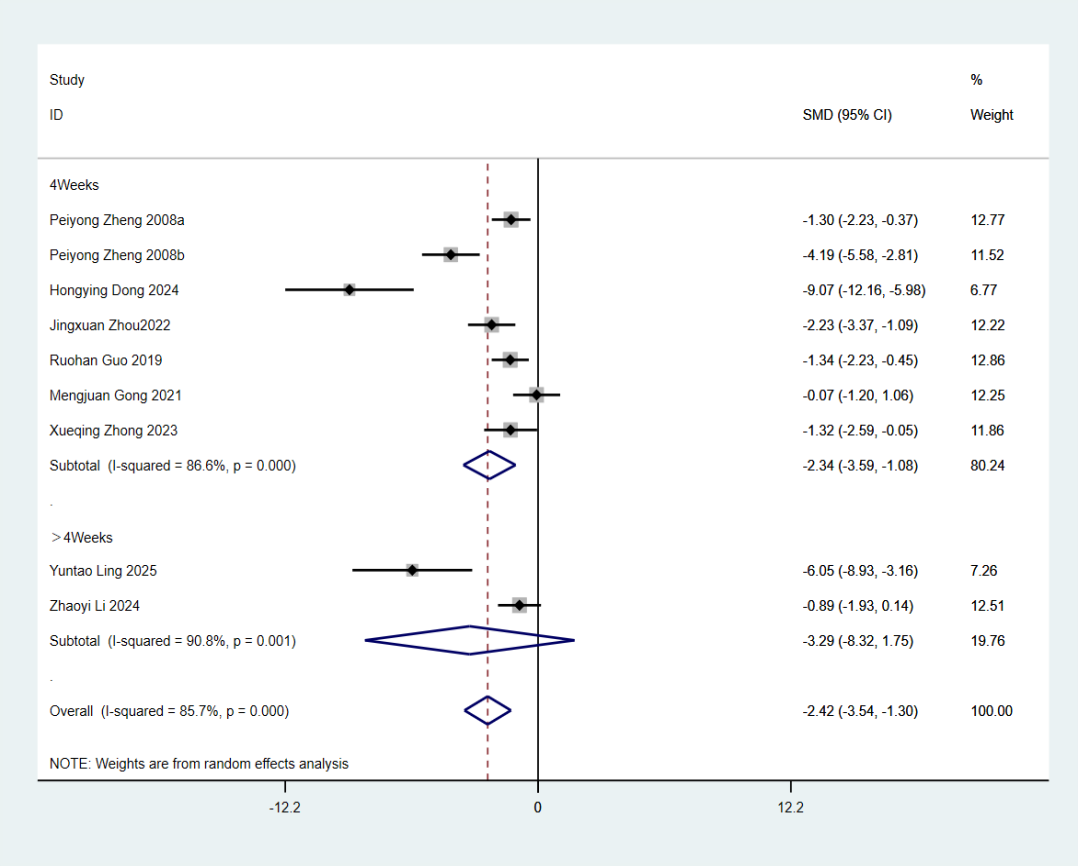


# Supplementary Figure 25. Subgroup analysis of liver TC based on intervention duration

#
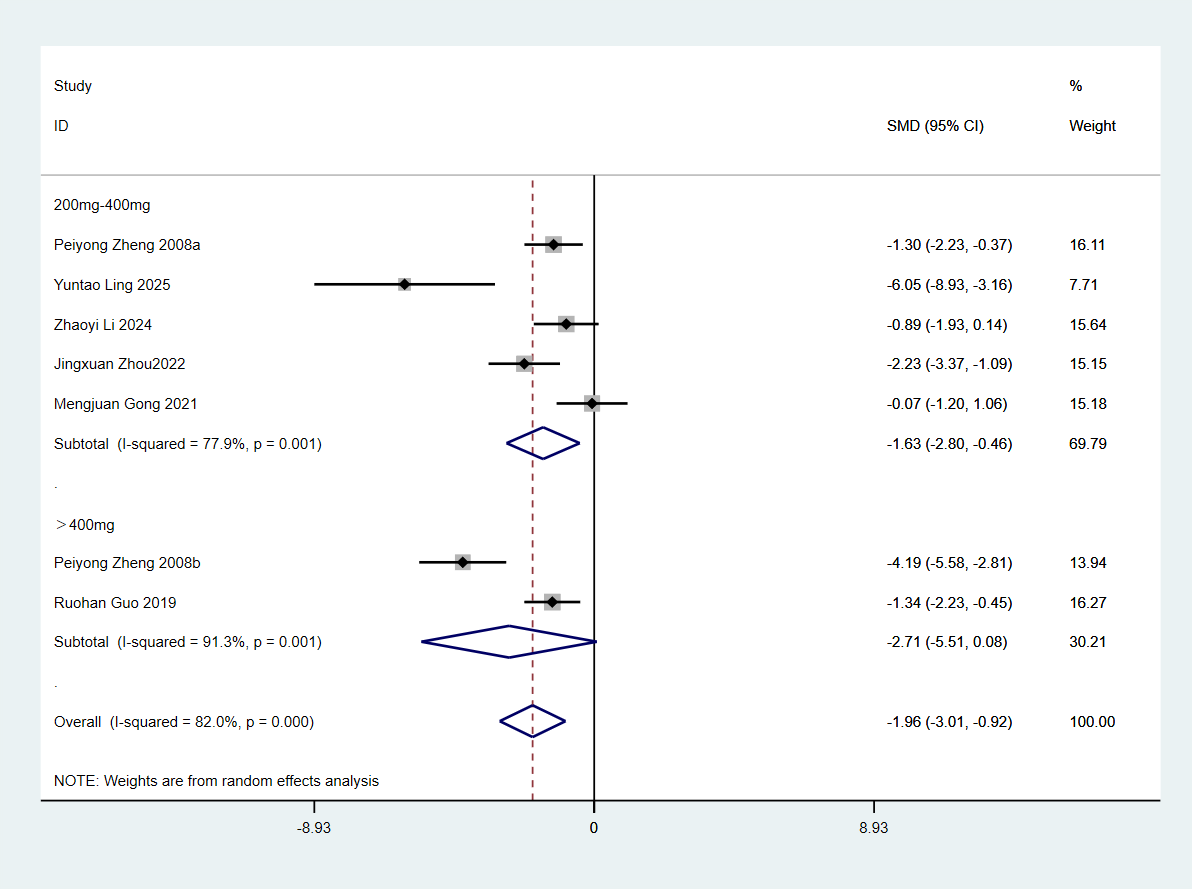


# Supplementary Figure 26. Subgroup analysis of liver TC based on dosage regimens of puerarin

#
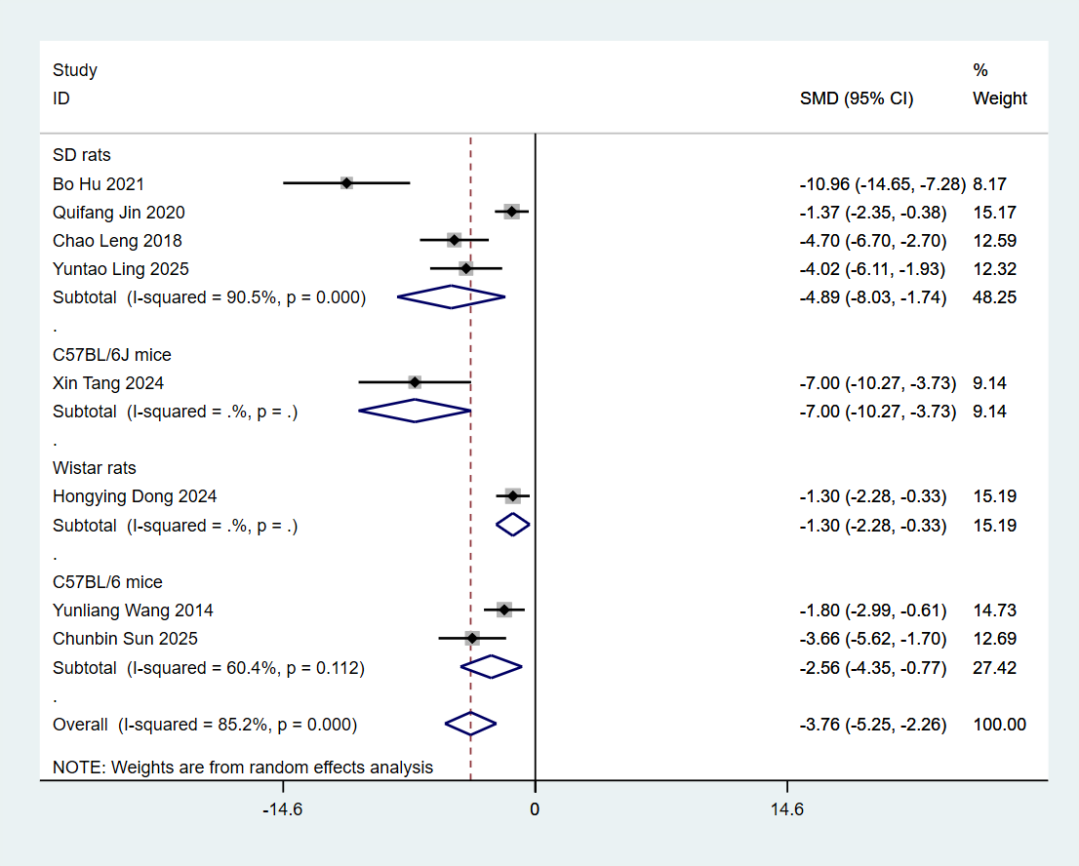


# Supplementary Figure 27. Subgroup analysis of LDL-C based on species of animal

#
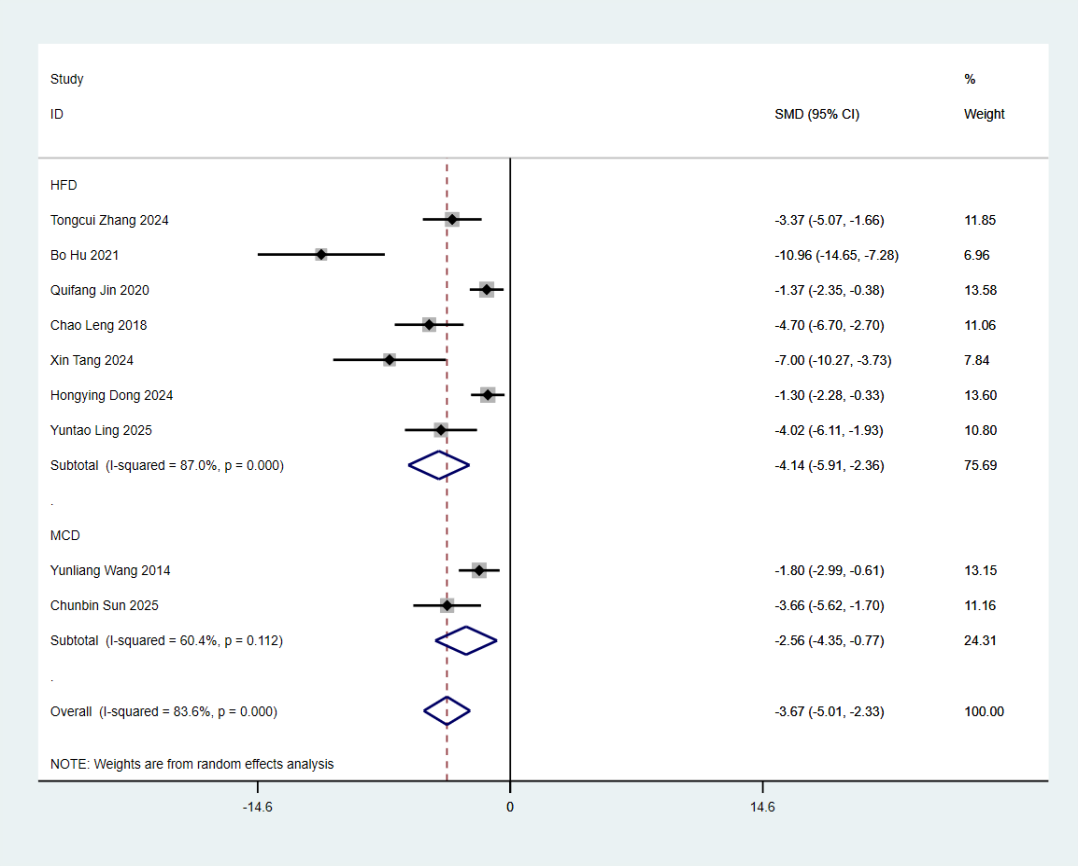


# Supplementary Figure 28. Subgroup analysis of LDL-C based on modeling methodologies

#
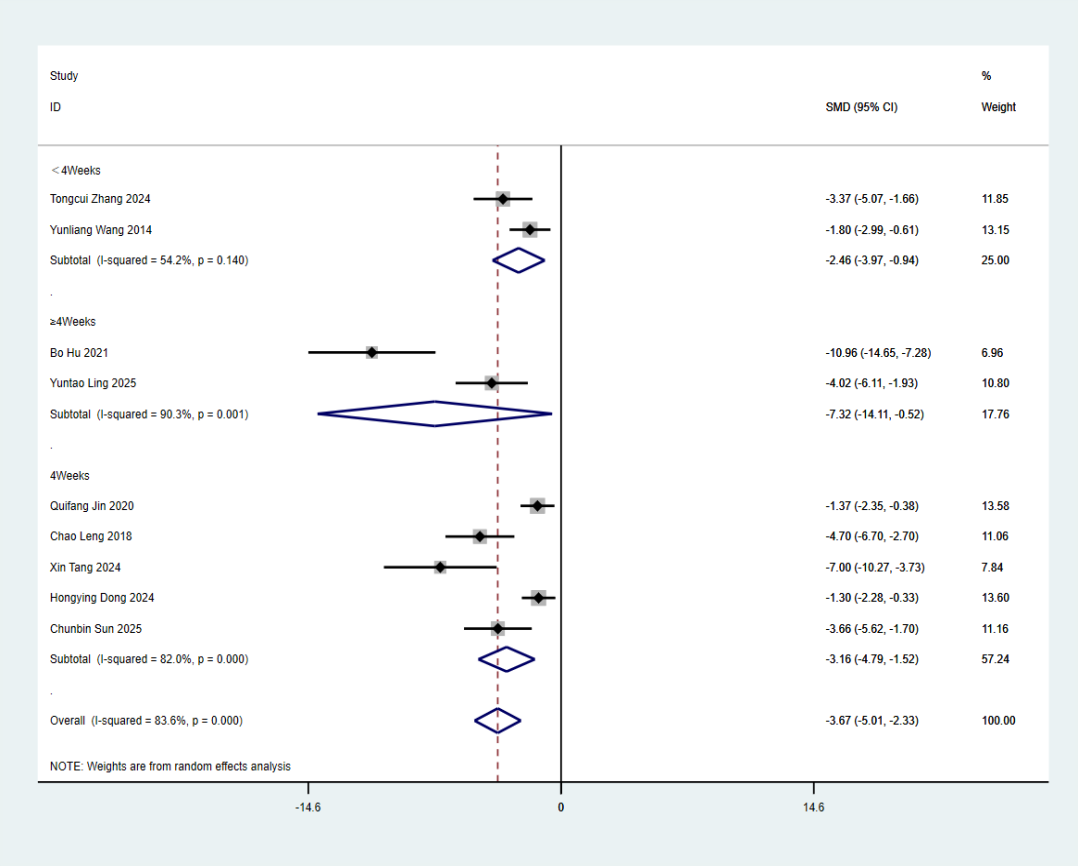


# Supplementary Figure 29. Subgroup analysis of LDL-C based on intervention duration

#
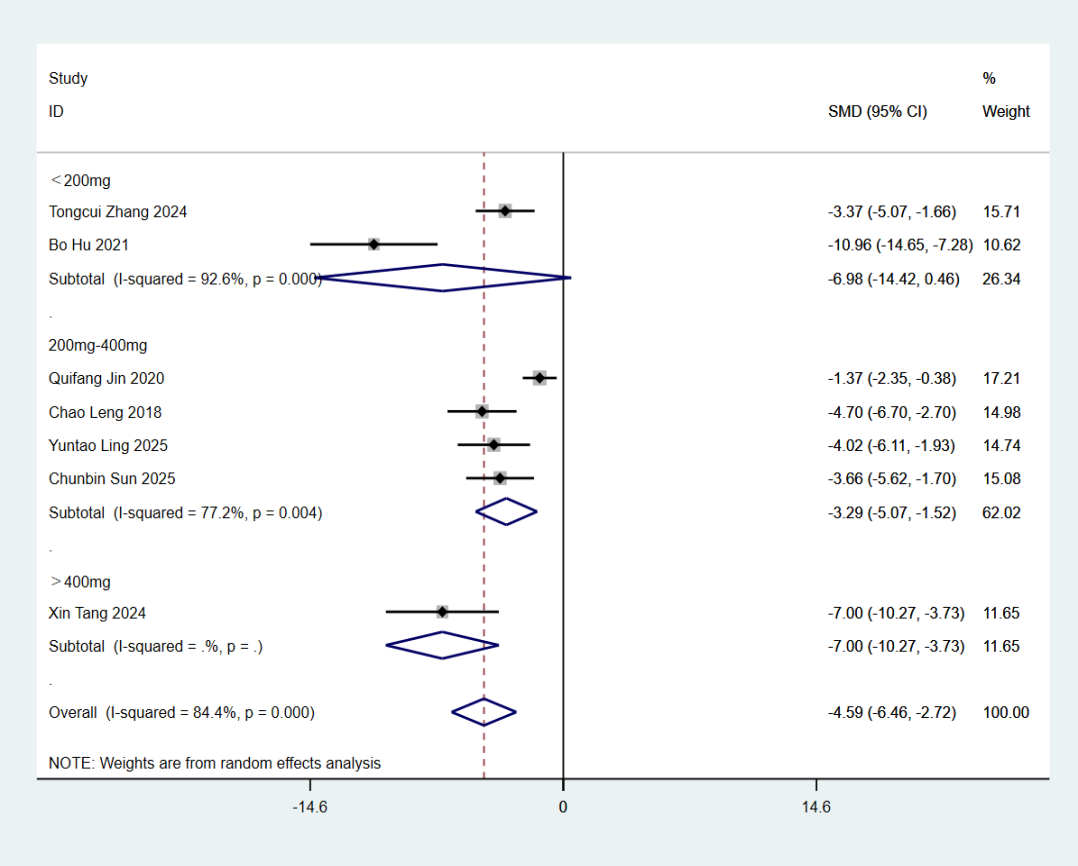


# Supplementary Figure 30. Subgroup analysis of LDL-C based on dosage regimens of puerarin

#
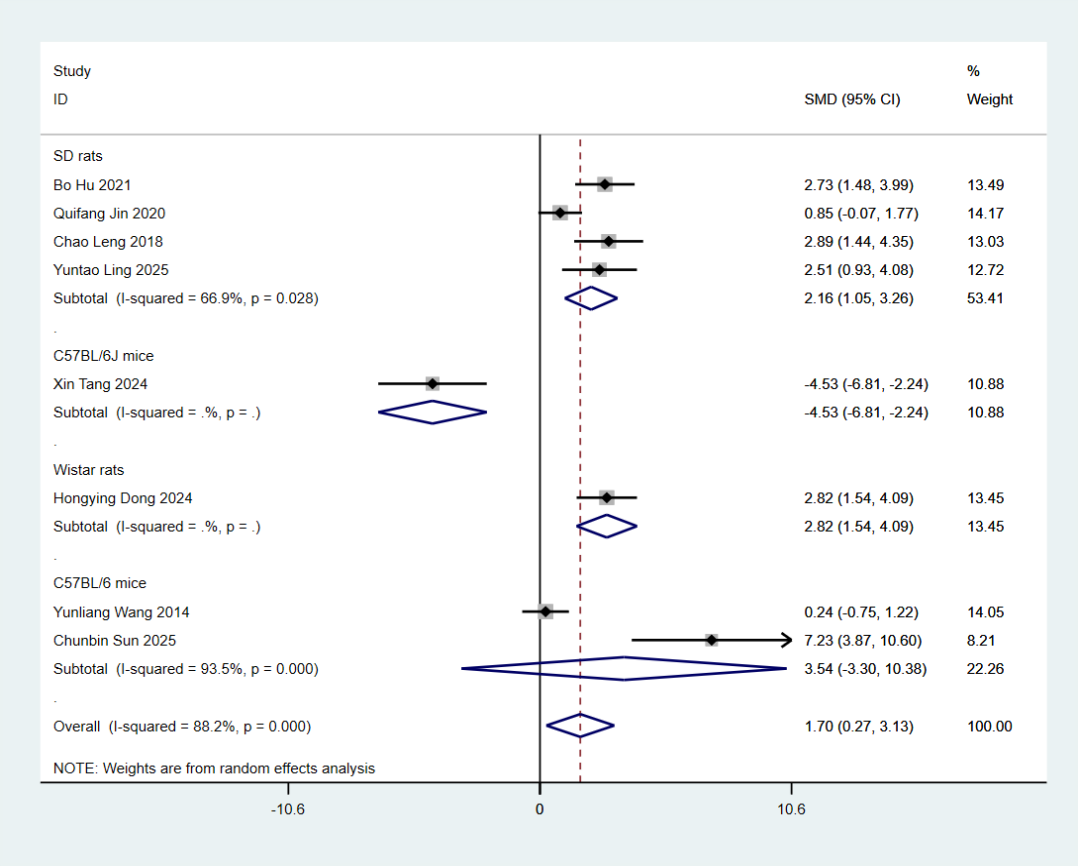


# Supplementary Figure 31. Subgroup analysis of HDL-C based on species of animal

#
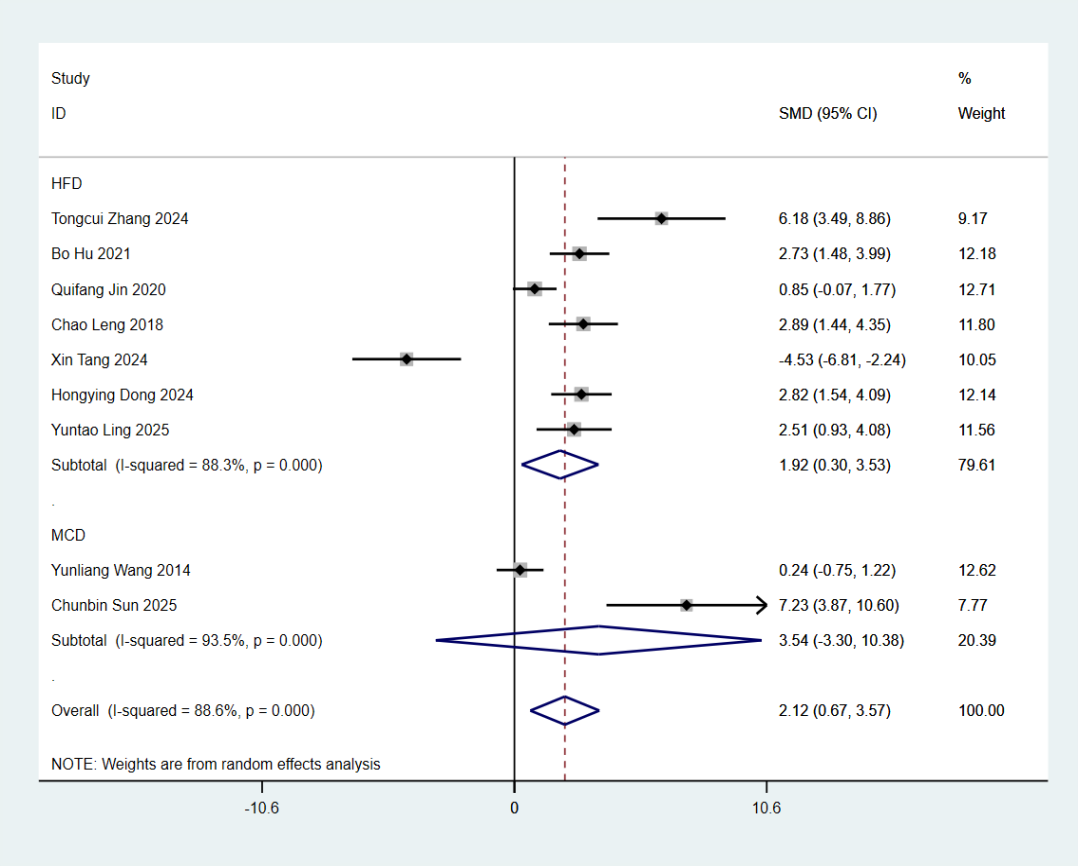


# Supplementary Figure 32. Subgroup analysis of HDL-C based on modeling methodologies

#
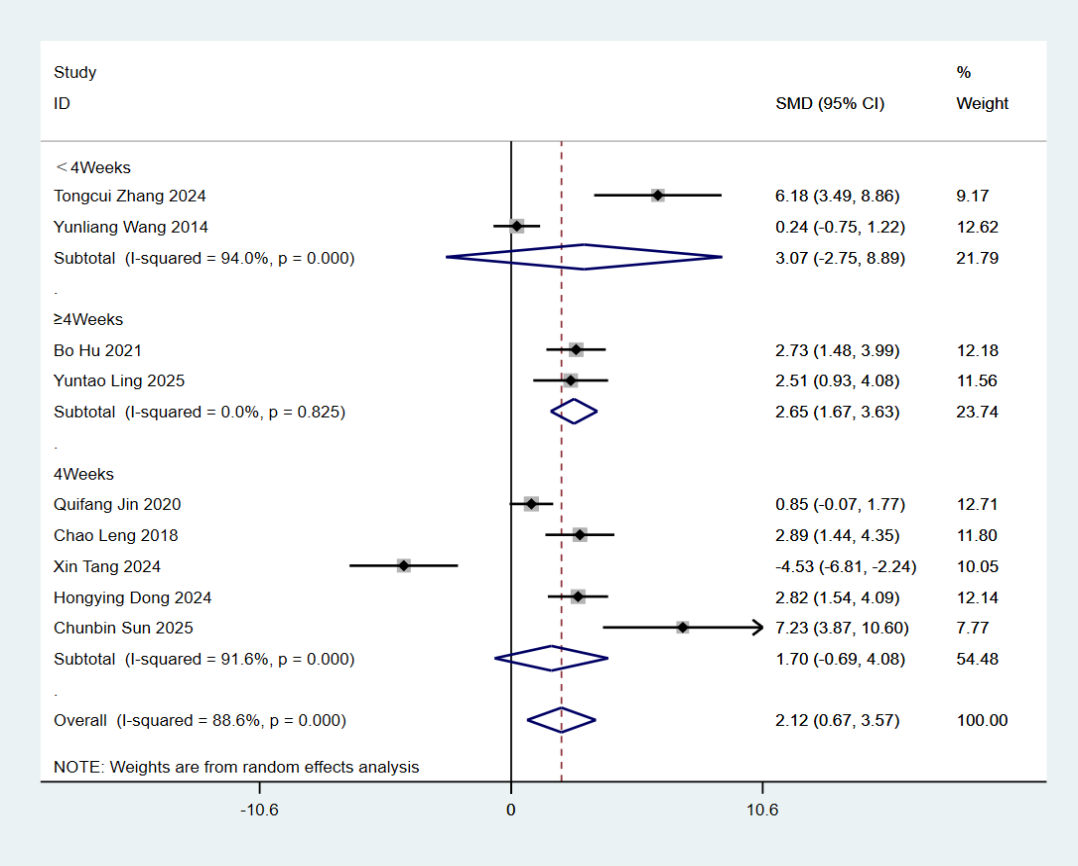


# Supplementary Figure 33. Subgroup analysis of HDL-C based on intervention duration

#
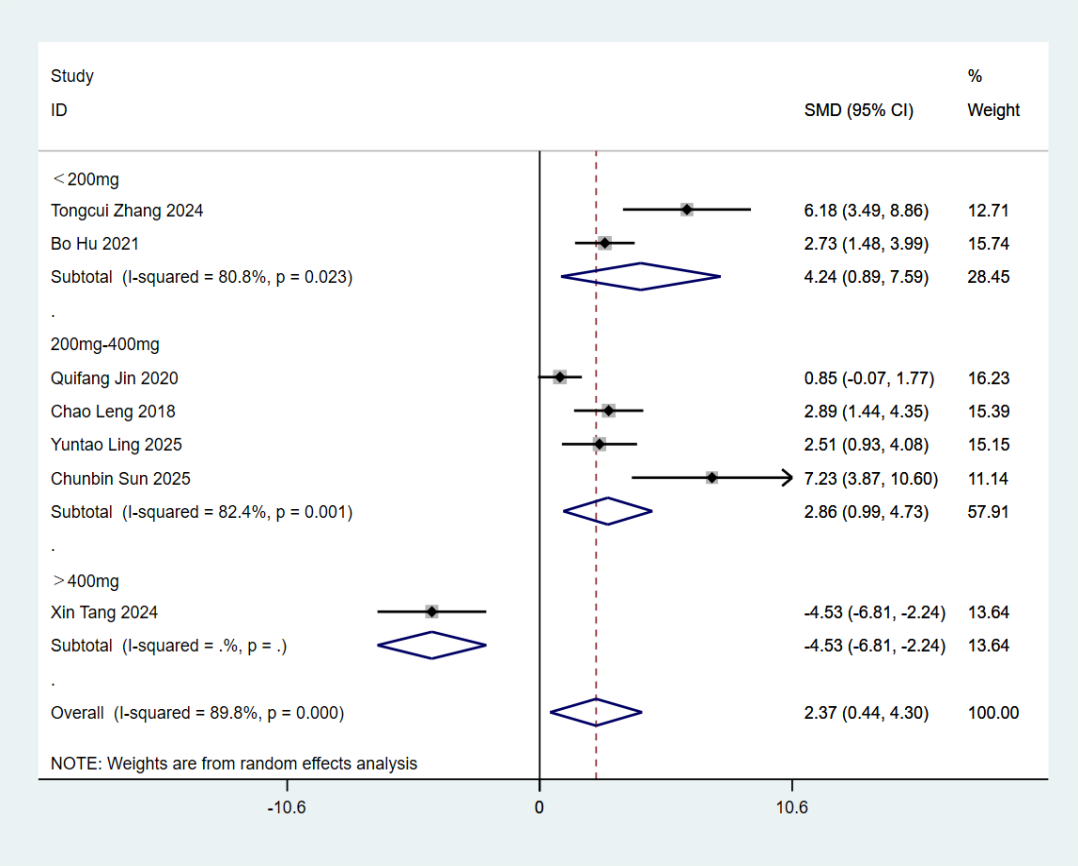


# Supplementary Figure 34. Subgroup analysis of HDL-C based on dosage regimens of puerarin

#
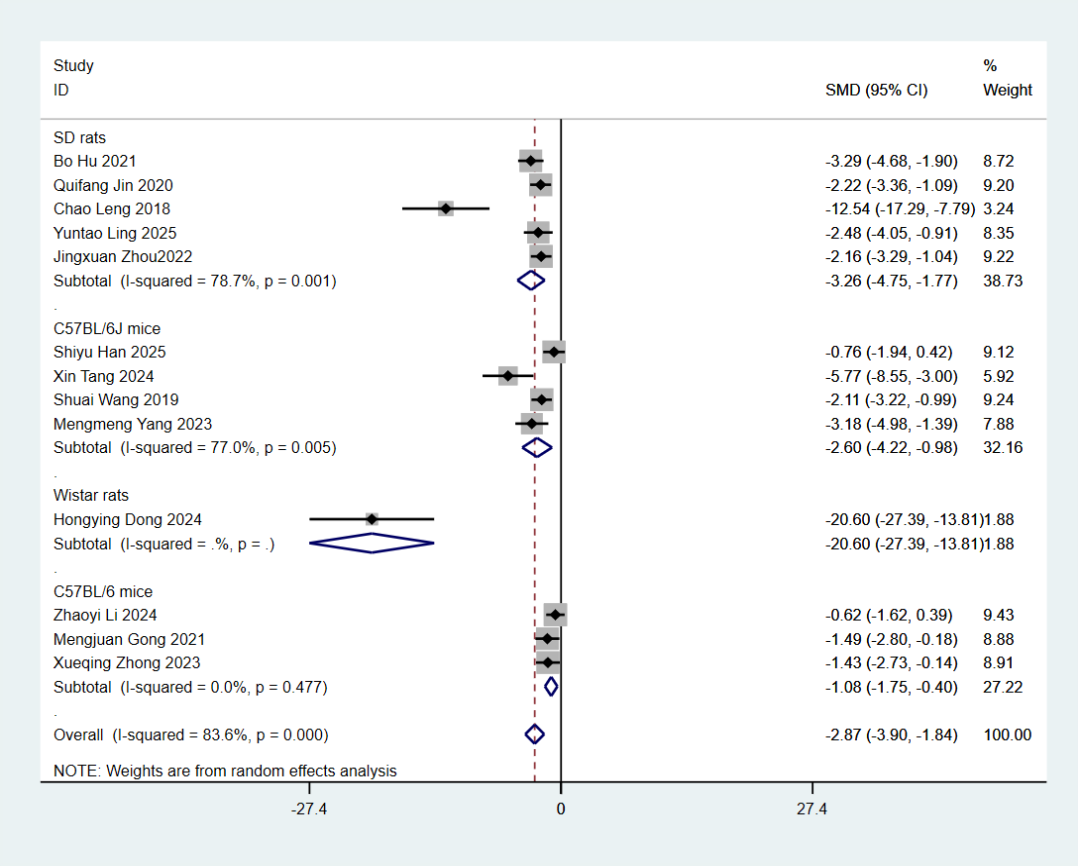


# Supplementary Figure 35. Subgroup analysis of AST based on species of animal

#
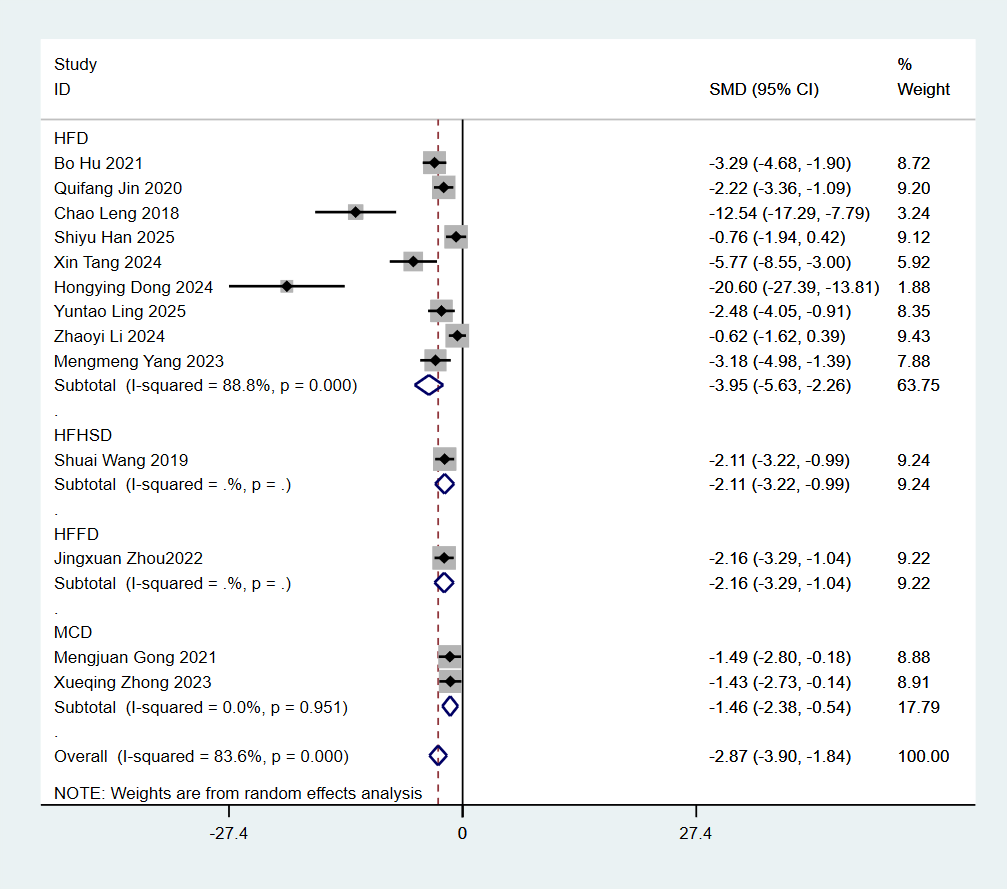


# Supplementary Figure 36. Subgroup analysis of AST based on modeling methodologies

#
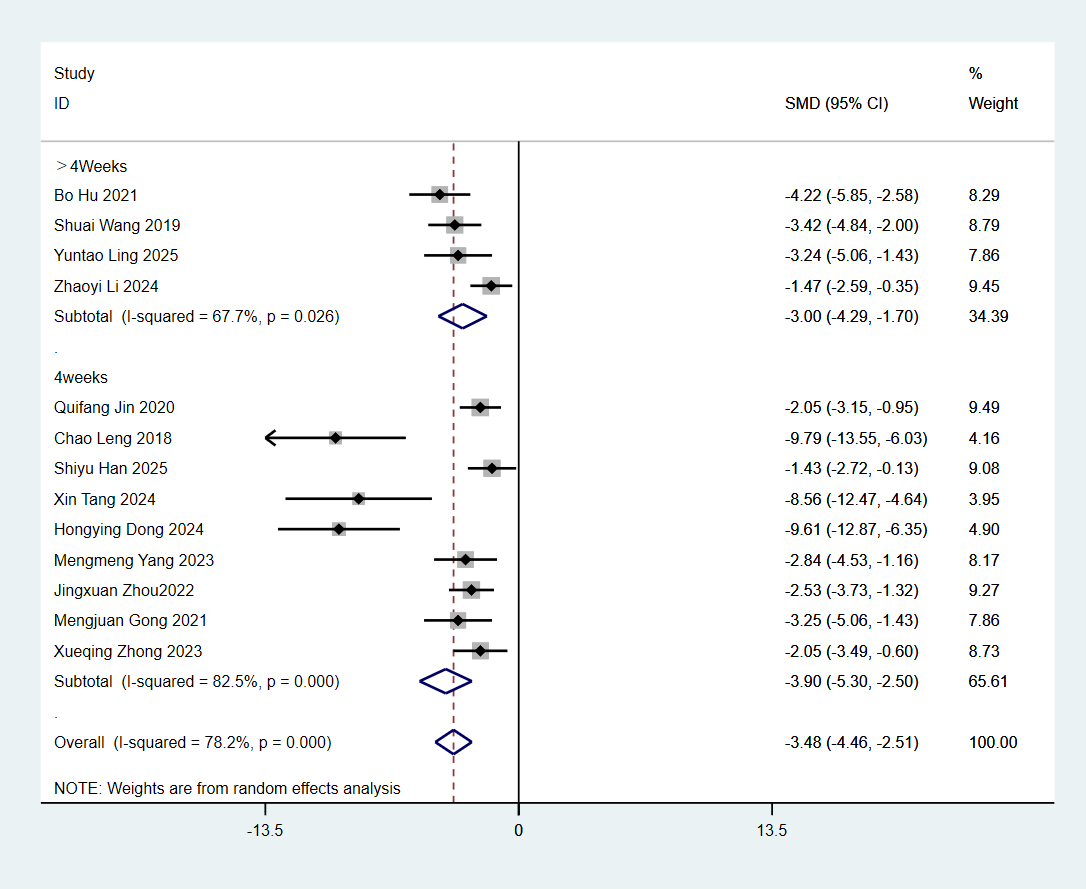


# Supplementary Figure 37. Subgroup analysis of AST based on intervention duration

#
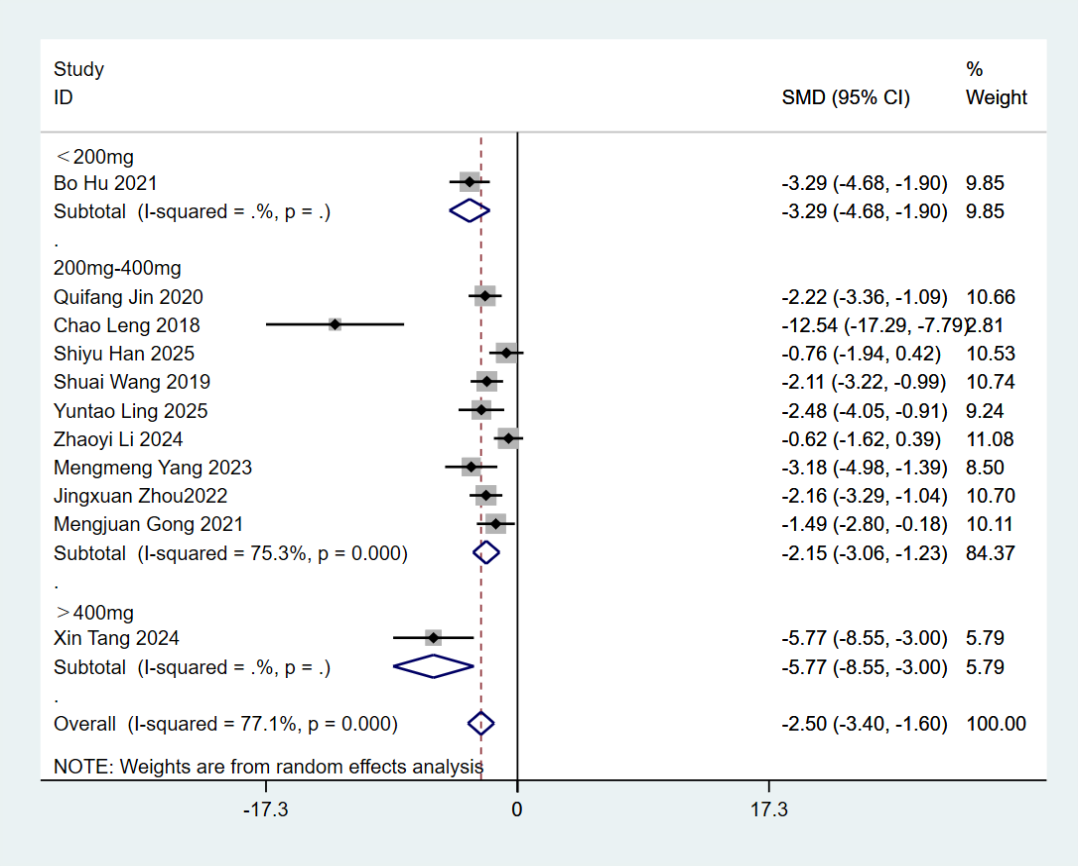


# Supplementary Figure 38. Subgroup analysis of AST based on dosage regimens of puerarin

#
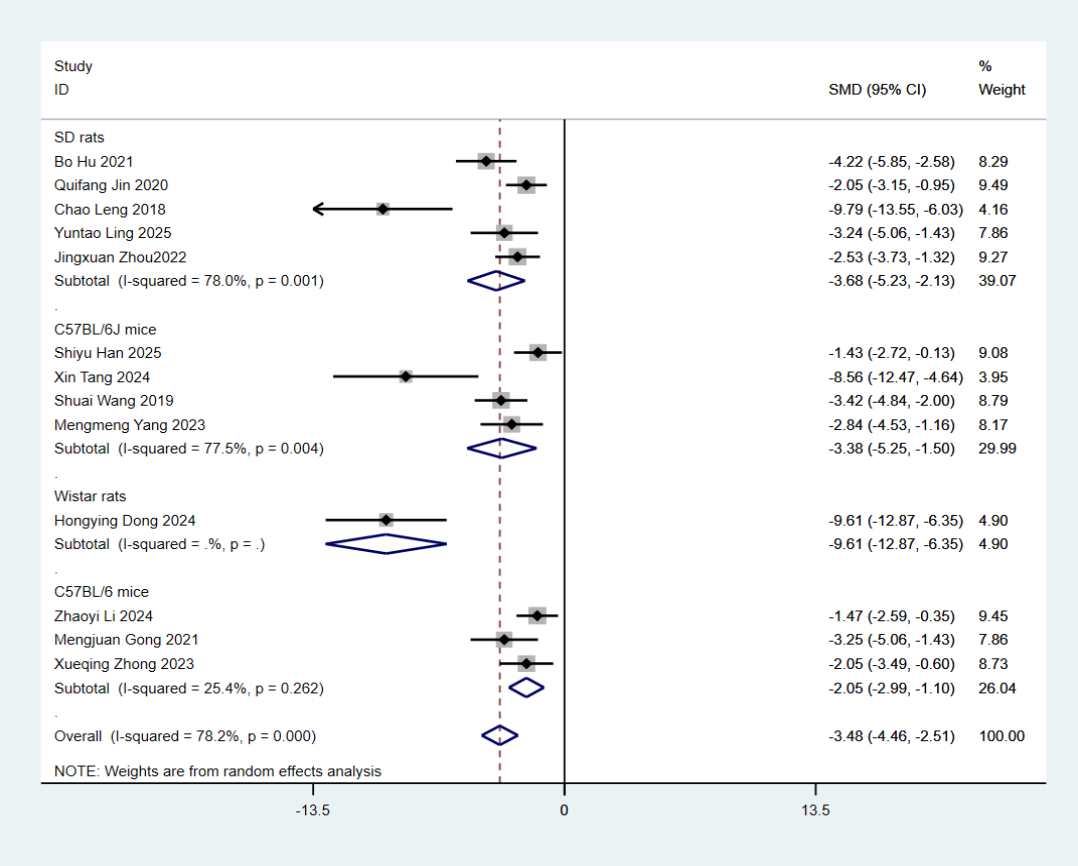


# Supplementary Figure 39. Subgroup analysis of ALT based on species of animal

#
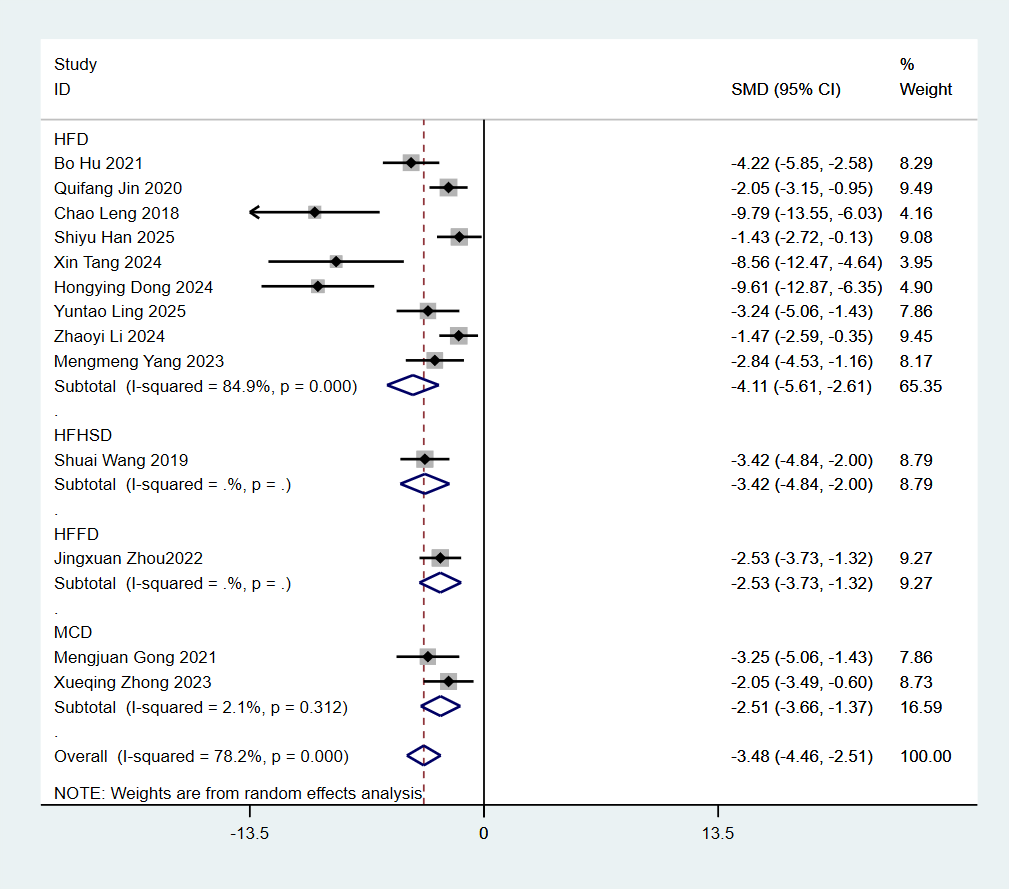


# Supplementary Figure 40. Subgroup analysis of ALT based on modeling methodologies

#
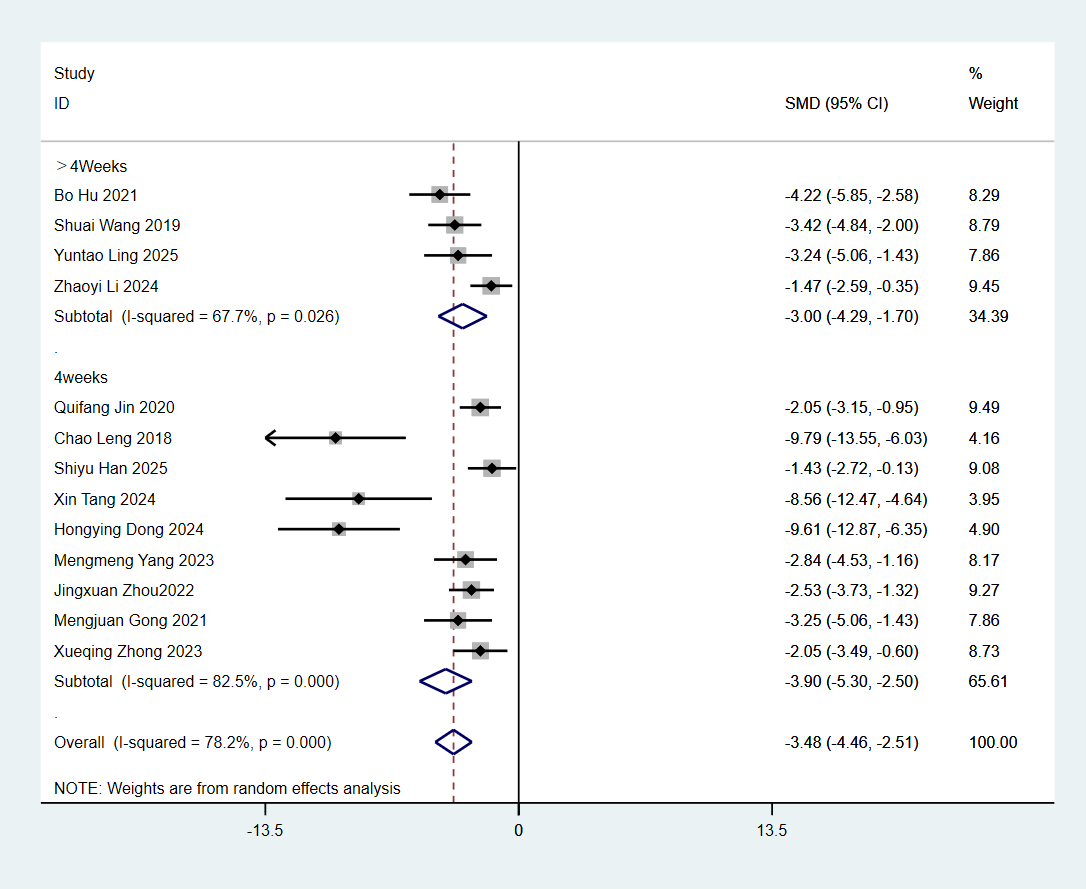


# Supplementary Figure 41. Subgroup analysis of ALT based on intervention duration

#
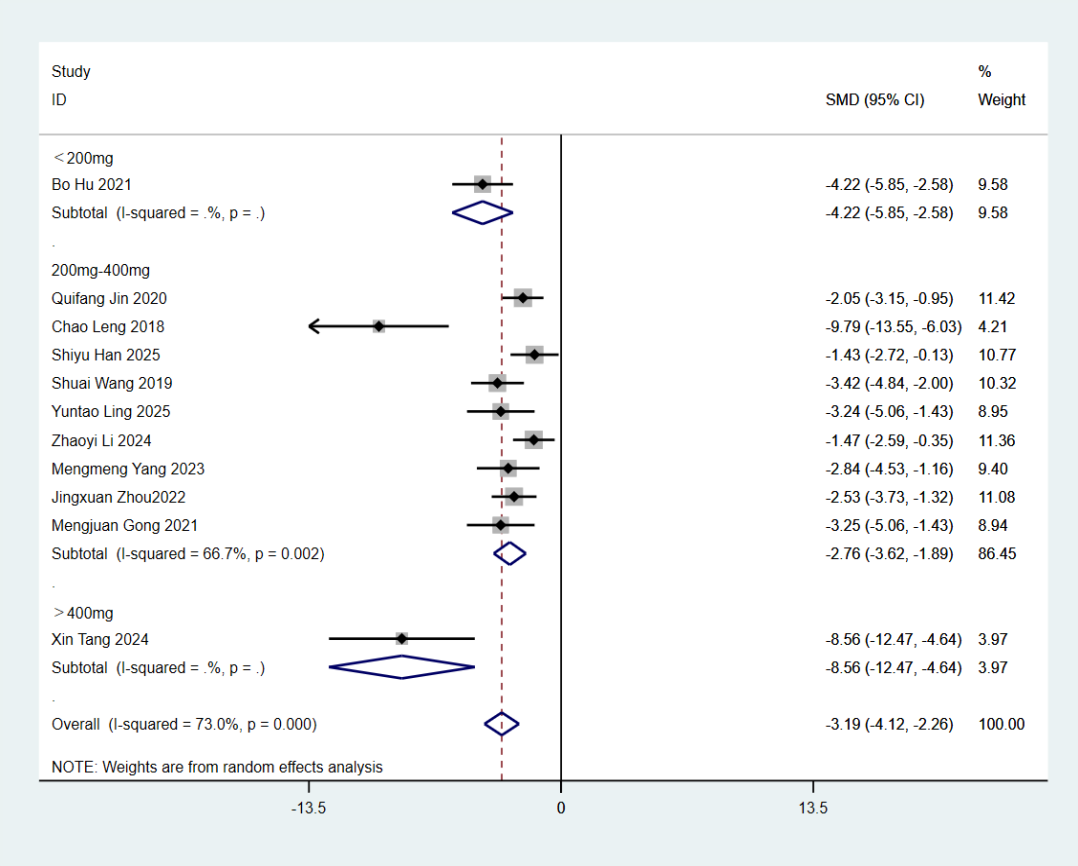


# Supplementary Figure 42. Subgroup analysis of ALT based on dosage regimens of puerarin
